# A key variant in the cis-regulatory element of flowering gene *Ghd8* associated with cold tolerance in rice

**DOI:** 10.1038/s41598-019-45794-9

**Published:** 2019-07-03

**Authors:** Peng Wang, Yin Xiong, Rong Gong, Ying Yang, Kai Fan, Sibin Yu

**Affiliations:** 1 National Key Laboratory of Crop Genetic Improvement, College of Plant Science and Technology, Huazhong Agricultural University, Wuhan, 430070 China; 2 Present Address: Department of Agronomy and Horticulture, University of Nebraska Lincoln, Lincoln, NE 68588-0660 USA

**Keywords:** Genetic variation, Natural variation in plants, Plant breeding

## Abstract

Variations in the gene promoter play critical roles in the evolution of important adaptive traits in crops, but direct links of the regulatory mutation to the adaptive change are not well understood. Here, we examine the nucleotide variations in the promoter region of a transcription factor (*Ghd8*) that control grain number, plant height and heading date in rice. We find that a dominant promoter type of subspecies *japonica* displayed a high activity for *Ghd8* expression in comparison with the one in *indica*. Transgenic analyses revealed that higher expression levels of *Ghd8* delayed heading date and enhanced cold tolerance in rice. Furthermore, a single-nucleotide polymorphism (T1279G) at the position −1279 bp that locates on the potential GA-responsive motif in the *Ghd8* promoter affected the expression of this gene. The 1279 T variant has elevated expression of *Ghd8*, thus conferring increased cold tolerance of rice seedlings. Nucleotide diversity analysis revealed that the approximately 25-kb genomic region surrounding *Ghd8* in the subspecies *japonica* was under significant selection pressure. Our findings demonstrate that the join effects of the regulatory and coding variants largely contribute to the divergence of *japonica* and *indica* and increase the adaptability of *japonica* to the cold environment.

## Introduction

Day length and temperature are two major environmental factors that shape plant growth and development and affect the flowering time and photoperiod sensitivity that determines the seasonal and regional adaptation of crops. Rice (*Oryza sativa* L.) is the staple food for approximately half of the world’s population. Improvements in its productivity are an important way to meet the need of the continuously increasing human population. The high yield potential in rice is usually associated with a long growth time, which is the basic requirement for developing enough vegetative source to support the large sink of the reproductive organ. As a short-day plant, rice flowers more rapidly when the day-length becomes shorter and heading more difficult when the day-length grows longer. Rice is planted worldwide, mostly in Asia area that have a wide latitude range with changing photoperiod conditions, from long-day (LD) conditions to short-day (SD) conditions. This wide expansion of rice throughout complicated geographic environments suggests that the rice genome experiences profound changes, with several key genomic regions being under natural selection for adaption.

The natural variation of genes controlling adaptive traits, such as flowering time, has been extensively studied. In cultivated rice, the variation of florigen *Heading date 3a* (*Hd3a*) expression has been found to be highly correlated with the diversification of flowering time^1^. *Hd3a* expression might be partly linked to the combination of allelic variation in the Heading Date 1 (HD1) protein and to the divergent expression levels of *Early heading date 1* (*Ehd1*)^1^. In addition, natural nucleotide variations in several flowering time inhibitors, such as *PRR37*, *Ghd7*, and *Ghd8/DTH8/LHD1*, and flowering time accelerators (*Ehd1*, *Ehd4*, *Hd3a*, *OsELF3/Hd17/Ef7*, and *DTH2)* show a strong association with flowering time and contribute to the wide distribution of rice cultivation at various latitudes^2–12^. However, most of the important natural variations in rice that determine the function of genes are located in the coding region^1,10,11,13–15^. The role of the variation in the gene promoter region that modulates the function to cope with changing environmental conditions is elusive. Given that the flowering genes have the characteristics of accurate spatiotemporal expression and plasticity in response to environmental signals^16,17^, variations in both the promoter and coding region of a gene might combine in an optimal form to affect the adaption ability, which has currently been rarely reported in rice.

Nuclear factor Y (NF-Y) is a ubiquitous CCAAT-box binding transcription factor formed by NF-YA, NF-YB and NF-YC subunits^18–20^. NF-Y has been reported to regulate downstream gene expression to improve plant tolerance to abiotic and biotic stresses^21^. NF-Y has also been identified as a flowering time regulator in plants and plays a pivotal role in regulating diverse aspects of plant growth and development^22^. In particular, the subunits NF-YB have a function on flowering time, which has been well studied. NF-YB is called HAP3 (heme activator protein 3) or CBF-A (CCAAT-binding factor A) in yeast and animals^22^. *Ghd8* was identified as a transcription factor belonging to NF-YB. It was cloned as a major quantitative trait locus (QTL) controlling heading date, plant height and grain productivity in rice. It acts as a flowering inhibitor in LD conditions and an activator in SD conditions^8,23,24^. Recently, it has been reported that GHD8 regulates flowering time through interacting with HD1 and other NF-Y/HAP family members such as OsHAP2 and OsHAP5. The GHD8-HD1-OsHAP5 complex directly binds the CORE2 element on the 5′UTR region of florigen *Hd3a* to affect the flowering time^25,26^. Although the mechanism of *Ghd8* in controlling flowering time is well elucidated, the contribution of the cis-regulatory variations of *Ghd8* to rice adaptation to the environments such as adverse temperature condition and unfavorable day-length in the changing climate is not clear.

To study whether natural variations of *Ghd8* exhibit an important determinant function in rice adaptation, we conducted sequence analysis of the entire *Ghd8* gene, including the promoter region and coding region, in a panel of 198 rice germplasm accessions. Several haplotypes in the promoter region and coding region of *Ghd8* were observed in this population. Two dominant haplotypes of the *Ghd8* promoter had a significant difference in the transcription level. Transgenic tests in rice demonstrated that the expression level of *Ghd8* was significantly associated with flowering time and cold tolerance. Moreover, a single nucleotide polymorphism (SNP) in the promoter region of *Ghd8* was found to be involved the transcriptional change. Our results demonstrate that the cis-regulatory variation of the *Ghd8* promoter modulating its transcription level confers a selective advantage and plays a critical role in adapting rice to a wide range of environments.

## Results

### Nucleotide variation of *Ghd8*

Previous studies have shown that several variants occur in the coding region of *Ghd8* that alter the function of flowering time or heading date^8,13,23^. To understand the nucleotide variation of the promoter region and its relationship with the coding region, we sequenced a 3.8-kb region of *Ghd8* in a rice collection of 198 accessions (Table S1). The sequence comparison revealed a total of 41 variants identified across the 3.8-kb *Ghd8* region, showing extensive nucleotide variations in both the promoter and coding regions of *Ghd8* in the rice collection.

**Figure 1:**
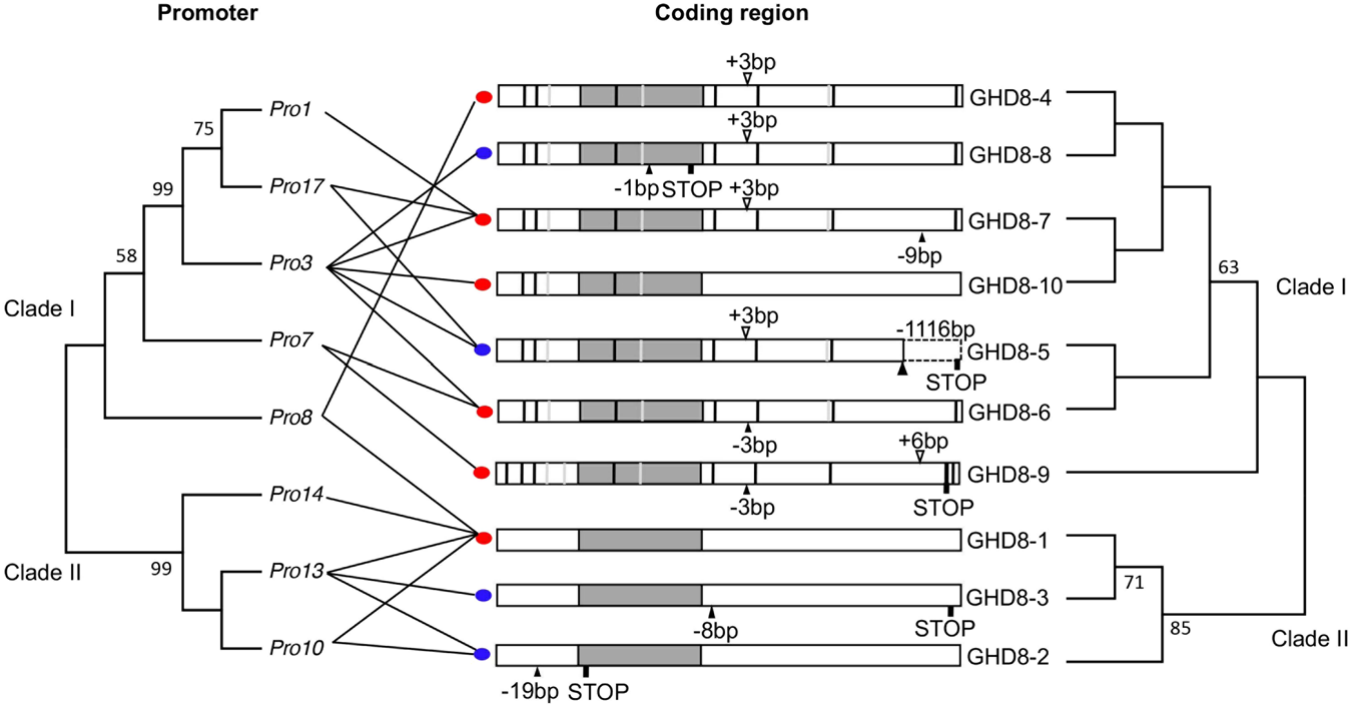
Phylogeny of the coding and promoter regions, showing two main clusters of the nucleotide variants in the promoter region (left) and coding region of Ghd8 (right). The relatedness of the major haplotypes was inferred by using the UPGMA method. The bootstrap values >50% (500 replicates) are indicated next to the branches. Ten *Ghd8* alleles based on the coding region variation are presented in the middle panel. The red and blue dots next to the coding region indicate the functional and loss-of-function alleles, respectively, based on the variation/mutation in the coding region and on previous reports. Open and closed arrowheads represent insertions and deletions, and the number denotes base pairs of the insertions or deletions compared to *Ghd8-1*. The black and gray vertical lines in the boxes indicate the nonsynonymous and silent SNPs, respectively. ‘STOP’ represents a premature stop codon.

Sixteen nucleotide variants were detected in the coding region of *Ghd8*, which classified the 198 accessions into 10 haplotypes (Fig. 1, Fig. S1). These coding haplotypes were divided into two clades (clade I and clade II) corresponding well to the subspecies *indica* and *japonica*. Seven haplotypes, GHD8-4, GHD8-5, GHD8-6, GHD8-7, GHD8-8, GHD8–9 and GHD8–10, were in clade I, and four haplotypes, including GHD8-1, GHD8-2 and GHD8-3, belonged to clade II (Fig. 1). In clade II, 90% of accessions shared the GHD8-1 haplotype^23^. In parallel, clade I was dominated by three haplotypes GHD8-5, GHD8-7 and GHD8-8, with respective frequencies of 24%, 15% and 18% in the 198 accessions (Table S1, Fig. S1). Notably, some haplotypes, such as GHD8-2, GHD8-3, GHD8-5, and GHD8-8, were reported to be nonfunctional, due to a frame-shift or stop codon occurring that caused a truncated or premature protein (Fig. 1), while GHD8-4, GHD8-6, GHD8-7 and GHD8–9 had a strong function^13,23^.

There were 25 variants detected in the promoter region of *Ghd8* in the 198 accessions. A phylogenetic analysis of the variants in the promoter region revealed that eight major haplotypes (minor frequency >0.02, or n ≥3) in the rice collection were identified and were also divided into the two clades as well (Fig. 1). Pro3 from clade I (*indica* type) and Pro13 from clade II (*japonica* type) were two dominant haplotypes (Table S1), existing in 50% and 24% of the rice collection, respectively.

A strong linkage disequilibrium (p < 0.0001, R^2^ > 0.9) was observed among the nucleotide variations in the promoter and coding regions of *Ghd8*. In particular, the strongest linkages existed between the dominant promoter haplotype Pro13 and the coding haplotype GHD8-1, and between Pro3 and GHD8-6 (Fig. 1, Table S2). These results suggest that the nucleotide variations in the promoter affecting the expression might be directly linked to the functional *Ghd8* alleles.

### *Ghd8* transcriptionally regulates flowering time

**Figure 2:**
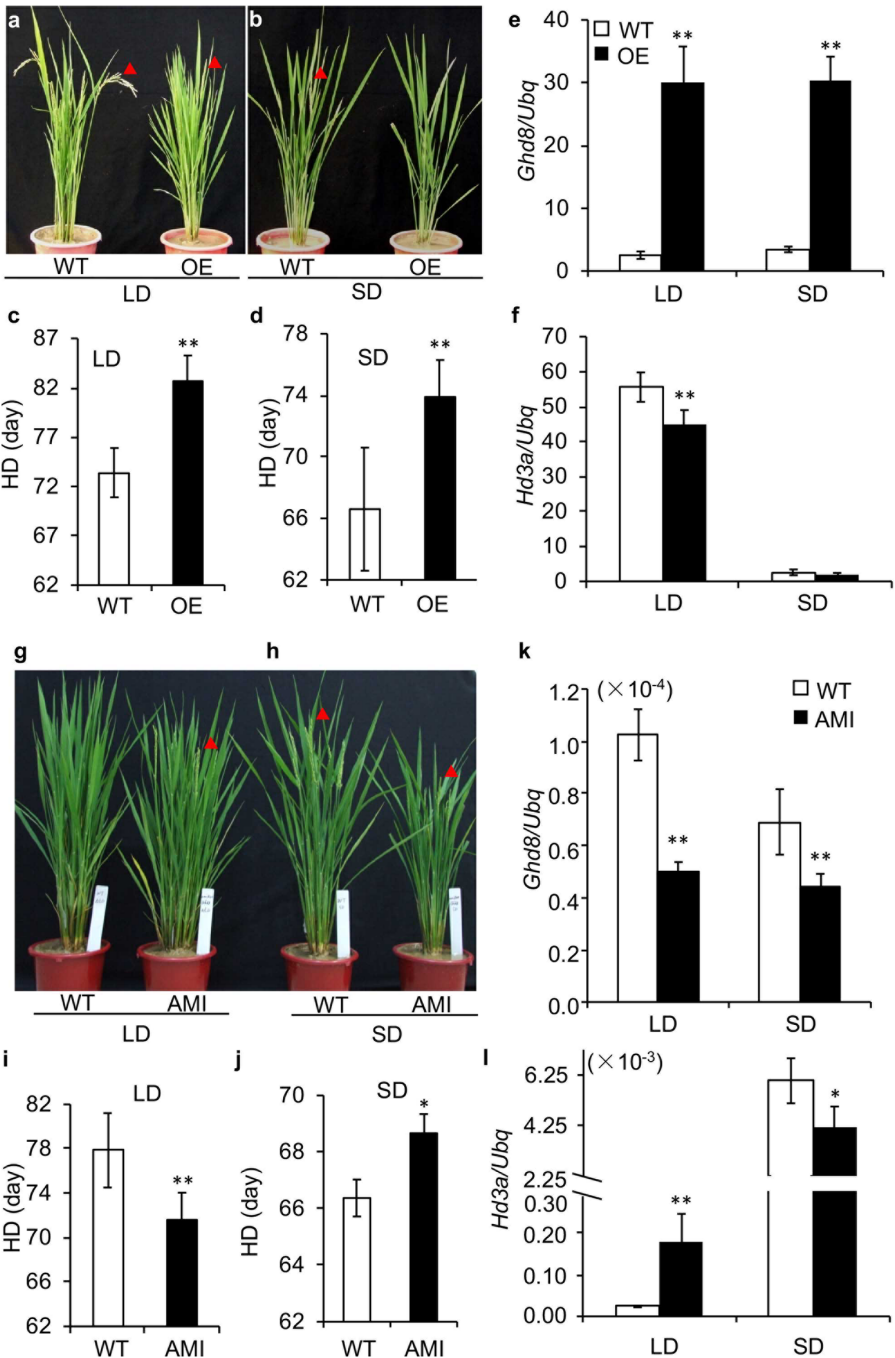
Altered expression of *Ghd8* affects rice heading date. (**a**–**d**) Phenotype performances of wild type (WT) and *Ghd8* overexpression line (OE) under LD conditions (14.5 h light: 9.5 h dark) (**a**) and SD conditions (9 h light: 15 h dark) (**b**). Arrows indicate the first emerging panicle. Heading date of WT and OE in LD (**c**) and SD (**d**) conditions. (**e**,**f**) The expression of *Ghd8* (**e**) and *Hd3a* (**f**). (**g**–**j**) Performances of WT and artificial microRNA line (AMI) of *Ghd8* in LD (**g**) and SD (**h**) conditions. Heading date of WT and AMI plants under LD (**i**) and SD (**j**) conditions. (**k**,**1**) The expression of *Ghd8* (**k**) and *Hd3a* (**l**) in WT and AMI plants. Asterisks ** and * represent significant difference compared to WT by *t*-test at p < 0.01 and p < 0.05 levels, respectively. Error bars indicate the mean ± SD, with three to five replicates.

To investigate whether the transcriptional changes of *Ghd8* are highly related to the flowering time, we generated overexpression lines of *Ghd8* (OE) with a *ubiquitin* promoter to drive the *Ghd8* allele from Nipponbare (designated as *Ghd8-1*), which is functional^23^. The OE lines showed delayed flowering of 5–7 days compared to wild type (WT) Nipponbare under both LD (14.5 h light: 9.5 h dark) (Fig. 2a,c) and SD conditions (9 h light: 15 h dark) (Fig. 2b,d). Consistent with the delayed flowering, the expression of *Ghd8* was 10−15 fold higher than that in the WT, and the downstream flowering gene *Hd3a* was significantly downregulated in the OE lines under both of the photoperiod conditions (Fig. 2e,f).

In addition, higher expression levels of *Ghd8* causing a delayed of flowering time was also found in a T-DNA mutant of *Ghd8* in which a 1.4-kb insertion fragment was detected from −170 bp to −1609 bp upstream of ATG in the *Ghd8-1* allele (Fig. S2). This insertion led to the overexpression of *Ghd8-1* that was 10–13 fold higher in the mutant compared to that in the WT. The overexpressing *Ghd8* caused a delayed (3 days) flowering time in the mutant lines (Fig. S2).

We also generated the amiRNA-*Ghd8* lines (AMI) in which the expression of *Ghd8* was repressed (Fig. 2g–l). The knock-down *Ghd8* in the amiRNA lines caused a dual phenotype. Compared to Nipponbare, the flowering time of AMI was advanced by approximately six days under LD conditions but was delayed by three days under SD conditions (Fig. 2g–j). Consistent with the earlier flowering time in LD conditions, the expression of the downstream gene *Hd3a* was induced in AMI (Fig. 2k,l). However, the expression of *Hd3a* was reduced in AMI under SD conditions, which matched the delayed flowering time in the short-day conditions (Fig. 2l). These results indicate that the *Ghd8-1* is functional allele and its expression levels are highly associated with flowering time in rice.

### Genetic variations modulate *Ghd8* expression

**Figure 3:**
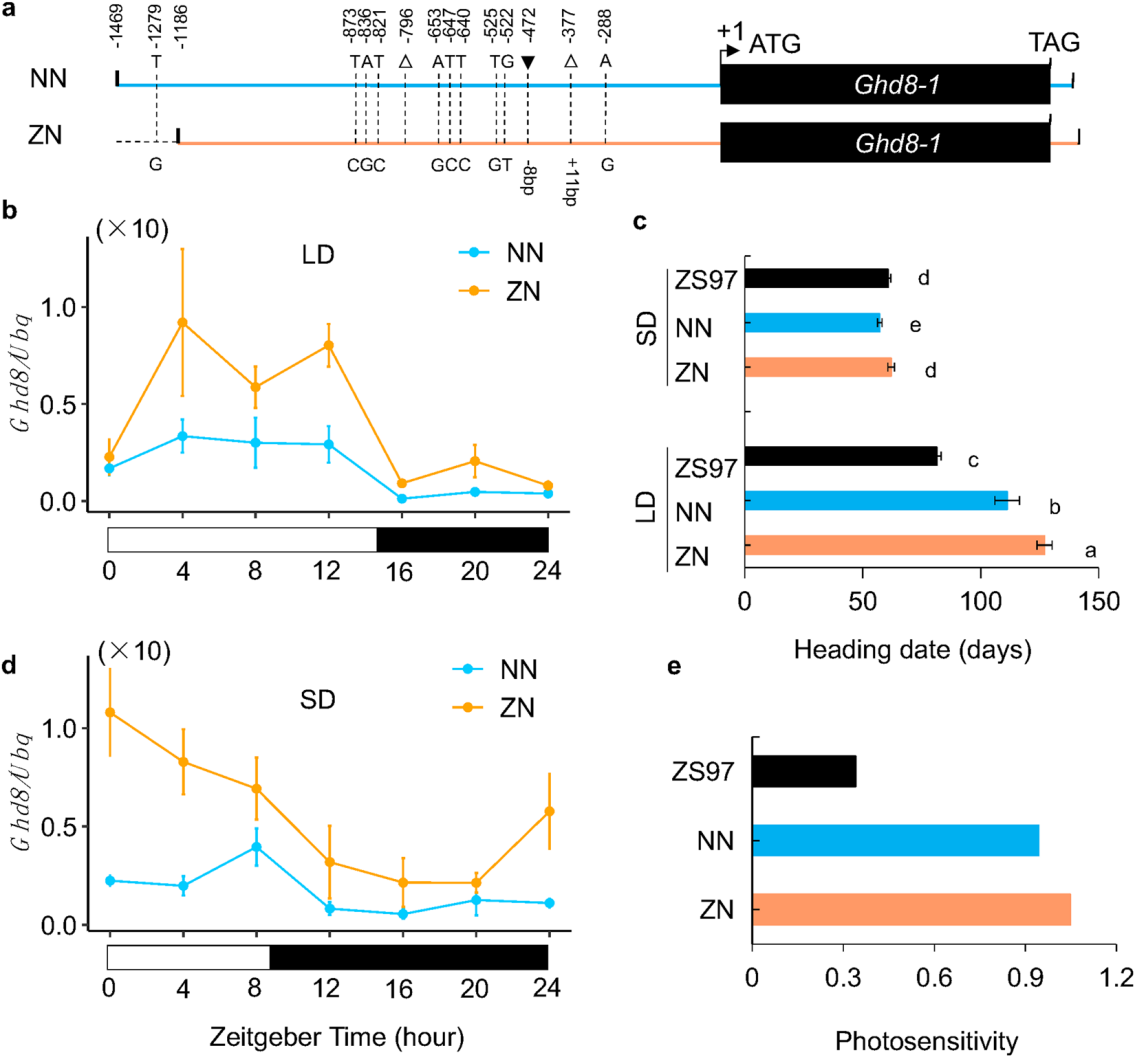
A negative cis-regulatory region of *Ghd8* regulates its expression level. (**a**) Schematic diagram of the *Ghd8-1* allele driven by two types of the *Ghd8* promoter, where the sequence variations are indicated. NN and ZN indicate the construct with the promoter type Pro13-1469 from Nipponbare and Pro3-1186 from ZS97, respectively. (**b**,**d**) Expression profiles in the NN and ZN transgenic plants under LD and SD conditions. The white and black bars represent light and dark periods, respectively. The zeitgeber times (ZT) on the axis are corresponding to the sampling times. Leaf samples were collected every 4 h from 35-day-old plants. (**c**) Heading date of the transgenic lines and ZS97 under SD and LD conditions. (**e**) Photosensitivity of the transgenic lines and ZS97. Different letters indicate significant difference among the lines by the Tukey’s HSD test at p < 0.05. Error bars indicate the mean ± SD with three replicates.

To test whether natural variations in the promoter contributed to the *Ghd8* expression that affected flowering time, we generated two constructs (named as ZN and NN) that harbored the common *Ghd8-1* driven by either a truncated promoter Pro3-1186 of Pro3 or a promoter Pro13-1469 of Pro13 (Fig. 3a). Higher expression levels of *Ghd8* were displayed in the ZN (Pro3-1186) transgenic lines than in the NN (Pro13-1469) transgenic lines (Fig. 3b,d). Moreover, ZN showed a significantly delayed flowering time compared to that of NN under both LD and SD conditions (Fig. 3c), which matched well with the overexpressing *Ghd8* delayed flowering time in the OE lines. In line with the flowering time delay and photosensitivity enhancement (Fig. 3e), reduced expression of downstream genes *Ehd1*, *Hd3a* and *RFT1* was observed in ZN (Fig. S3). Since both ZN and NN had the same *Ghd8-1* allele (Fig. 3a), the differences in flowering times between them should be a result of the functional variations in their promoters. These results indicate that the promoter region of *Ghd8* is associated with its differential expression, which plays a role in the flowering time of rice.

### SNP (T1279G) affects *Ghd8* expression

**Figure 4:**
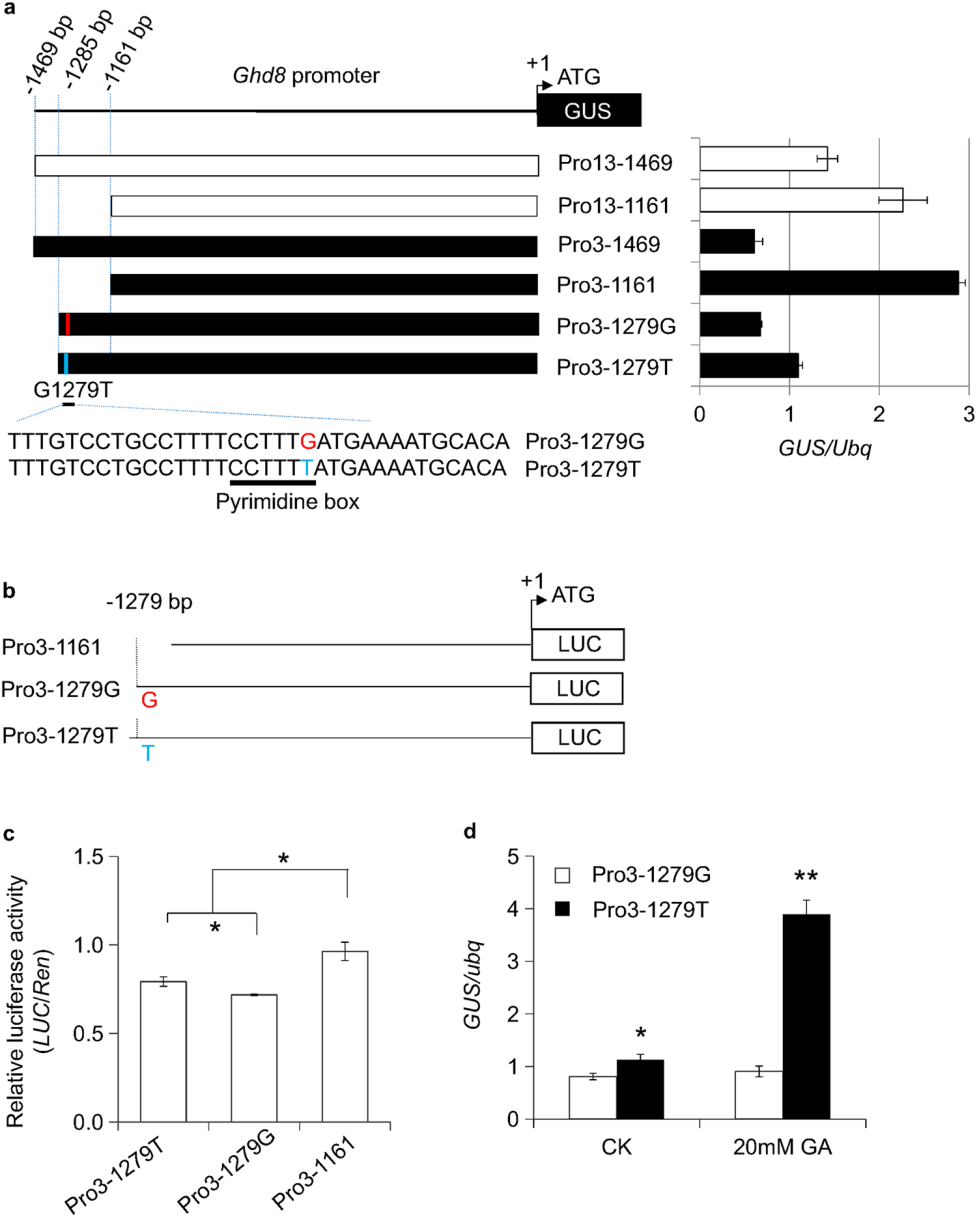
Expression of *GUS* and *LUC* driven by truncated *Ghd8* promoters. (**a**) Schematic diagram of the reporter gene *GUS* driven by the truncated promoters of *Ghd8* that were used to generate the transgenic *GUS* lines. Pro13-1161 represents the truncated promoter of *Ghd8* from Nipponbare (Pro13-1469). Pro3-1161 and Pro3-1279G indicate the truncated promoters from ZS97 (Pro3-1469). Pro3-1279T indicates a G replacement of T at the −1279 site of Pro3-1279G, where a predicted pyrimidine box is shown. The *GUS* expressions in 35-day-old seedling leaves of the corresponding transgenic lines, as indicted in the right panel. (**b**) Schematic diagram of constructs with the truncated promoters Pro3-1161, Pro3-1279G and Pro3-1279T for luciferase assays in rice protoplasts. (**c**) Relative firefly luciferase activity to Renilla luciferase activity of Pro3-1161, Pro3-1279G and Pro3-1279T. (**d**) *GUS* expression of Pro3-1279G and Pro3-1279T transgenic plants under the treatments of 0 mM (as CK) and 20 mM exogenous GA3. ** and * indicate significant differences among the indicated lines by ANOVA at p < 0.01 and p < 0.05 levels, respectively. Error bars indicate the mean ± SD with three replicates.

To determine which variants in the promoter play a key role in the regulation of *Ghd8*, we developed two more truncated promoter constructs Pro3-1161 and Pro13-1161 from Pro3-1469 and Pro13-1469, respectively. The expression of the *GUS* (β-glucuronidase) reporter driven by the truncated promoter Pro3-1161 showed a higher expression level than that driven by Pro3-1469. Likewise, the *GUS* expression by the truncated promoter Pro13-1161 was much higher than that by Pro13-1469 (Fig. 4a, Fig. S4). A higher expression level of *GUS* was observed in Pro3-1161 than in Pro13-1161. In contrast, *GUS* expression in the transgenic line driven by Pro3-1469 was two times lower than that in the Pro13-1469 transgenic line (Fig. 4a, Fig. S4). In addition, the GUS-staining results showed there was no difference in tissue-specific expression between the Pro3::GUS and Pro13::GUS transgenic lines (Fig. S5). There was no significant difference in *GUS* expression between the transgenic lines with Pro3-1469 and the other truncated promoter, Pro3-1279G (Fig. 4a). These results suggest that the distal region (−1285 to −1161 bp) has a negative cis-regulatory element reducing the expression of *Ghd8*.

Remarkably, only the SNP (T1279G) at site −1279 was found within the 125-bp segment of the distal region between Pro3 and Pro13. Transient expression assays with the additional site-mutated construct (Pro3-1279T), in which 1279 G was replaced by 1279 T (Fig. 4b), showed that the expression level of *GUS* in Pro3-1279T was significantly increased relative to Pro3-1279G and partially recovered up to 50% of the expression level in Pro13-1469 (Fig. 4a). Likewise, Pro3-1279T revealed a significantly higher luciferase activity in the rice protoplasts than that in Pro3-1279G but was significantly lower than that in Pro3-1161, which lacked the 125-bp segment (Fig. 4c). Thus, the variant (e.g., 1279 G) might negatively affect *Ghd8* expression regulation.

### *Ghd8* has a conserved function in *Arabidopsis*

**Figure 5:**
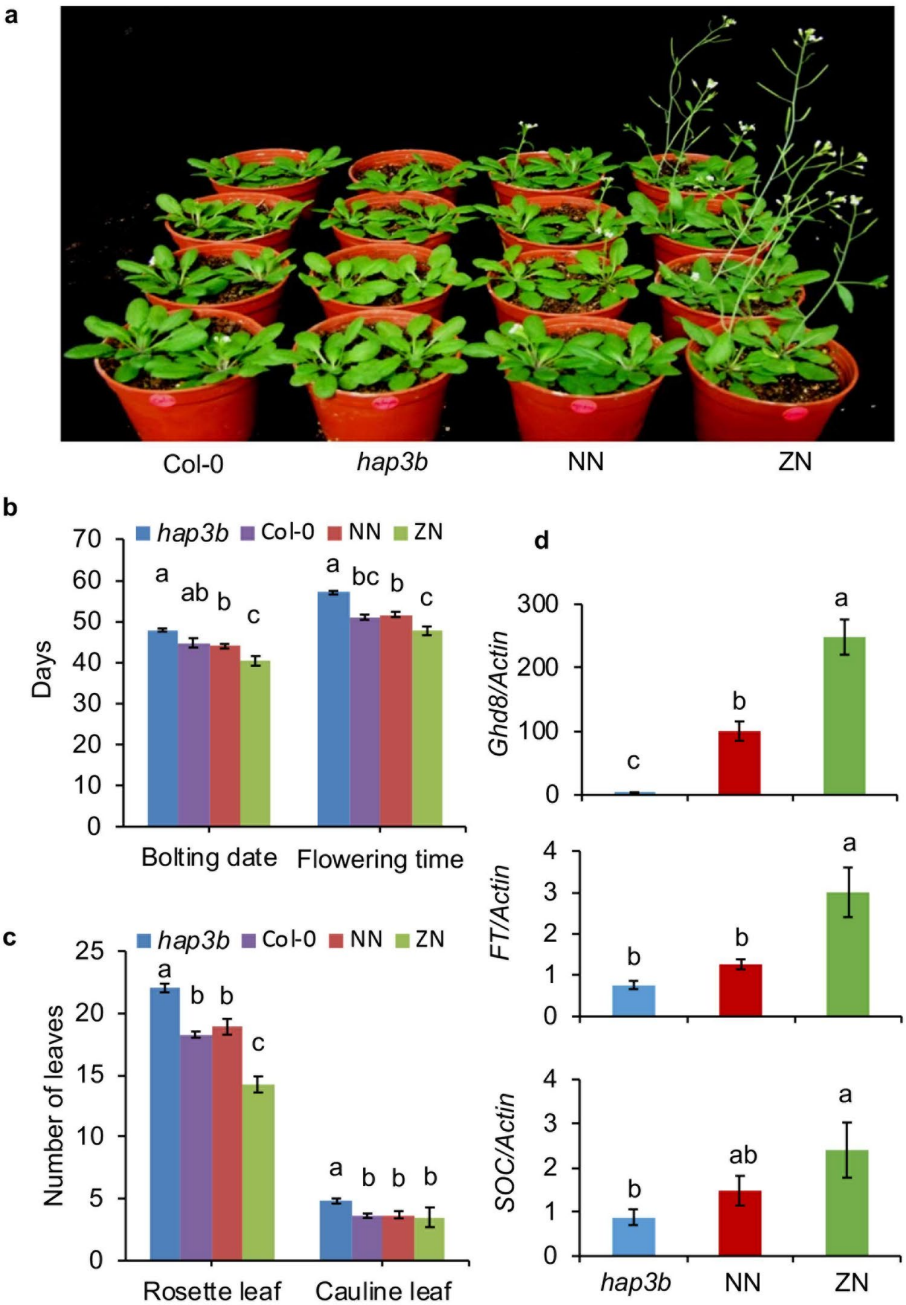
*Ghd8* rescues the late flowering of *hap3b* mutant in *Arabidopsis*. (**a**) Performance of wild-type Col-0, late flowering mutant *hap3b*, and transgenic plants generated with the NN and ZN constructs (four pots of each column belong to the same genotype) under LD conditions (16 h light: 8 h dark). (**b**) Bolting time and flowering time for the indicated genotypes as shown in (**a**). (**c**) Number of rosette leaves and cauline leaves scored at the maturity stage. At least 10 plants for each indicated genotype were recorded, and the error bars represent the mean ± SE. (**d**) Expression of *Ghd8*, *FT* and *SOC* in the *hap3b* mutant, NN and ZN transgenic plants in *Arabidopsis* under LD conditions. Different letters above the bar indicate significant differences by Tukey’s HSD test at p < 0.05.

GHD8 protein in rice shares a highly conserved domain with the early flowering time gene *Hap3B* in *Arabidopsis*. Our previous results showed that overexpression of *Ghd8*, driven by 35 S, significantly accelerated the flowering time in *Arabidopsis* Col-0^23^. To validate whether the rice promoter normally drives *Ghd8* expressed in *Arabidopsis*, we also introduced the rice constructs ZN and NN into the late-flowering mutant *hap3b* in *Arabidopsis*. The transgenic test results showed that both the ZN and NN transgenic lines could positively recover the mutant phenotype in bolting date, flowering time, and rosette and cauline leaf number to the level of wild type Col-0 (Fig. 5a–c). Furthermore, the ZN transgenic lines flowered much earlier than the NN lines. Similar to the expression pattern in rice, much higher *Ghd8* expression levels were found in the ZN lines relative to that of NN in *Arabidopsis* (Fig. 5d). As expected, the downstream florigen gene *FT* and the flowering integrator *SOC* were upregulated more profoundly in ZN than in NN (Fig. 5d). These results indicated that the rice *Ghd8-1* could rescue the late flowering time in the mutant *hap3b*, and the functional effect was regulated through the rice promoter. Therefore, *Ghd8* has a conserved function to control flowering time in *Arabidopsis* and rice, and its expression is functionally associated with the flowering time.

### High expression of *Ghd8* confers cold tolerance in rice

**Figure 6:**
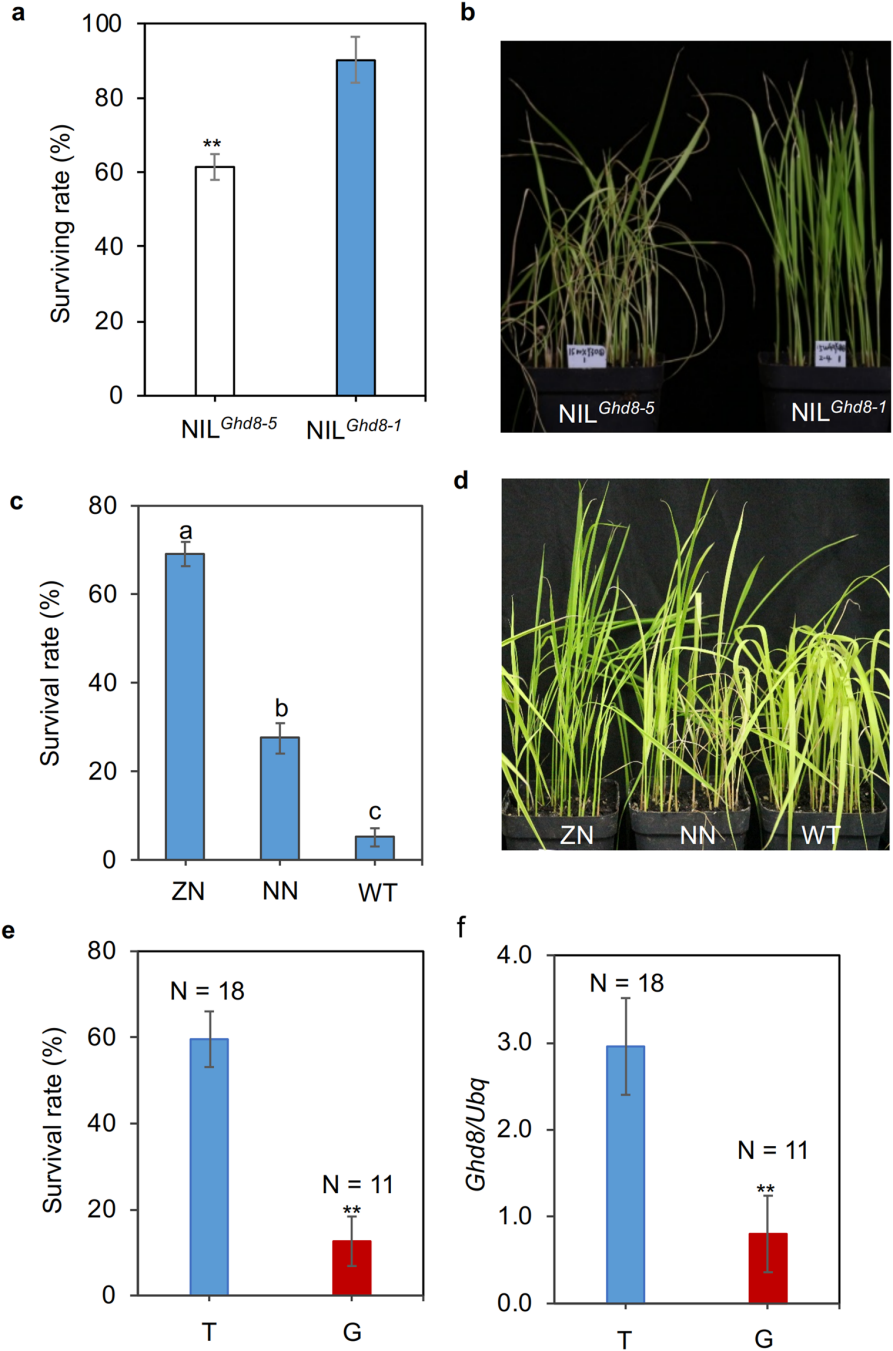
A higher expression level of *Ghd8* confers better cold tolerance in rice. (**a**) Difference in seedling survival rate between near-isogenic line (NIL) carrying the *Ghd8-1* alleles and *Ghd8-5* under cold treatment. (**b**) The performances of NIL^*Ghd8-1*^ and NIL^*Ghd8-5*^. (**c**) Survival rate of the ZN and NN transgenic lines and WT (ZS97) under cold stress. (**d**) The seedling performances of the indicated lines. (**e**) Comparison of survival rates of the accessions carrying the 1279 T variant and those carrying the 1279 G variant. N represents the number of the assayed rice accessions. (**f**) Expression level of *Ghd8* in the accessions carrying the T or G alleles as shown in (**e**). The error bars indicate the mean ± SE. Asterisks ** represent significant differences between the indicated lines at p < 0.01 by *t*-test; and different letters above the bar indicate significant differences by ANOVA at p < 0.05.

Low temperature is one of the major abiotic stresses limiting rice distribution in specific environments such as the high-latitude regions. To understand whether *Ghd8* regulates cold tolerance in rice, we developed two near-isogenic lines (NILs) within the common ZS97 background carrying two contrasting alleles: a nonfunctional allele *Ghd8-5* and a functional allele *Ghd8-1*. The NILs with different functional *Ghd8* alleles showed a significant difference in seedling survival rate after 4 °C treatment. NIL^*Ghd8-1*^ displayed an improved cold tolerance, with approximately 30% higher survival rate compared to that of NIL^*Ghd8-5*^ (Fig. 6a,b). Notably, in line with *Ghd8-1* affecting the flowering time, the average survival rate of the *Ghd8-1* transgenic line was 27% higher than that of ZS97. The average survival rate of the ZN transgenic line with the increased expression level of *Ghd8* was 40% higher than that of NN (Fig. 6c,d). Consistently, a high expression level of *Ghd8* conferring a strong cold tolerance in rice was also observed in the *Ghd8* overexpression line (Fig. S6).

**Figure 7:**
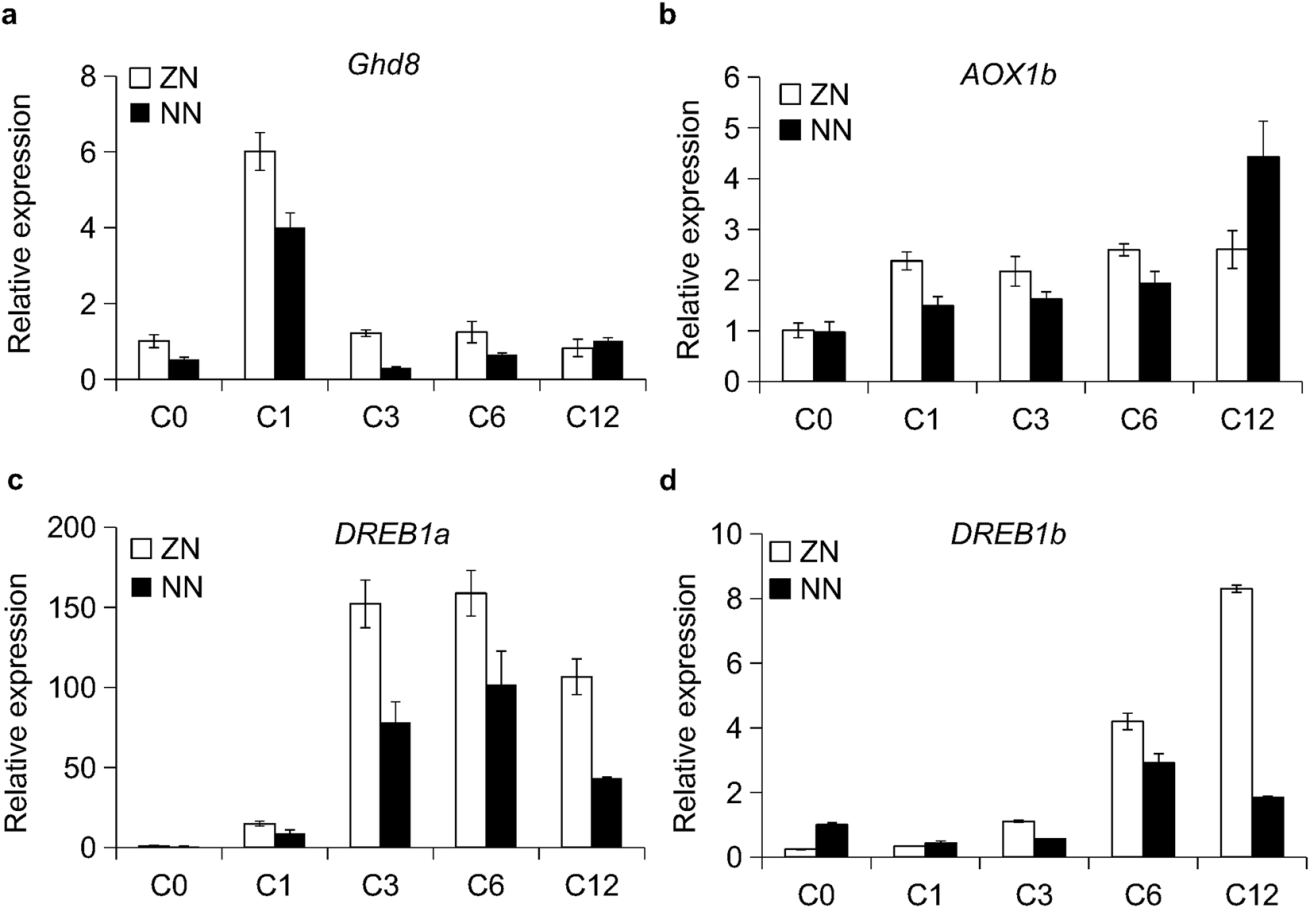
The expressions of *Ghd8* and several cold response genes were induced by cold treatment. (**a**–**d**) Expression profiles of *Ghd8* (**a**) and three cold response genes, *AOX1b* (b), *DREB1a* (**c**), and *DREB1b* (**d**), in the transgenic lines ZN and NN. C0, C1 and C12 represent 0, 1 and 12 h after cold treatment. The error bars indicate the mean ± SD with three replicates.

To understand how *Ghd8* responds to low temperature, we carried out an expression analysis of *Ghd8* in cold treatment using the ZN and NN transgenic lines in rice. Quantitative real-time PCR analyses showed that *Ghd8* expression in the transgenic lines of ZN and NN were all rapidly induced by six to eight times at 1 h after cold treatment, and ZN showed a continuously higher expression level of *Ghd8* than did NN up to to 6 h after cold treatment (Fig. 7a). In addition, several cold responsive genes, such as *AOX1b*, *DREB1a* and *DREB1b*^27,28^, were detected with a significant upregulation in both ZN and NN under the cold stress (Fig. 7b–d). Consistent with the high survival rate of ZN in the cold treatment, the cold responsive genes in ZN showed a significantly higher expression level than those in NN across all the surveyed time points (Fig. 7b–d). These results indicate that *Ghd8* is induced by low temperatures and is associated with cold tolerance through positively upregulating cold-response genes in rice.

The association of *Ghd8* with cold tolerance was also confirmed by analyzing data from the variant T1279G in natural rice cultivars. The rice accessions with the promoter haplotype carrying T1279G displayed a significant difference in the expression level of *Ghd8*, in which the 1279 T had a higher expression level than the 1279 G (Fig. 6f). These results support that the variant T1279G located in a negative regulatory element, is key to determining the expression level of *Ghd8*. Furthermore, the rice varieties with the promoter haplotype carrying 1279 T exhibited a significantly higher cold tolerance than those of the accessions carrying the 1279 G (Fig. 6e,f). Taken together, these results confirm that T1279G is a strong candidate for the causal regulatory variant of *Ghd8*, leading to differences in the cold tolerance of rice.

### Phytohormone GA might mediate *Ghd8* expression

To determine which conserved regulatory elements might be associated with the SNP T1279G, we analyzed the sequences within the 125-bp fragment surrounding the SNP using the software PLACE^29^. It predicted that the fragment contains one potential pyrimidine box with a CCTTT**T/G** motif that is involved in gibberellic acid (GA) responsiveness^30^. The SNP (T/G highlighted above) at position −1279 is located at the pyrimidine box (Fig. 4a). To test whether T1279G alters the GA responsiveness, we performed luciferase (*LUC*) transient assays in rice protoplasts. The assays showed that the relative activity of *LUC* by the construct Pro3-1279T was significantly higher than Pro3-1279G under the normal condition without GA treatment. Notably, an approximately twofold greater increase of *LUC* activity was observed in Pro3-1279T relative to Pro3-1279G when treated with 5 μM GA3, but it remained unchanged in Pro3-1279G (Fig. S7). The results imply that the GA response of the regulatory element was abolished when the CCTTTT motif was mutated. Furthermore, quantification of the *GUS* expression in leaf tissue driven by Pro3-1279T also exhibited a significantly stronger *GUS* expression level than that by Pro3-1279G, especially after 20 mM GA3 was applied to the *GUS* transgenic seedlings (Fig. 4d). These results suggest that the variant T1279G, located in the GA-responsive motif, might modulate the expression of *Ghd8*.

### Genome region encompassing *Ghd8* in *japonica* subspecies under selection

To gain insight into the divergent variants in the promoter that are linked to adaptive traits, we compared the nucleotide diversity between cultivated and wild rice and conducted neutrality tests for the sequences across a 100-kb genomic region flanking *Ghd8*. Diversity analyses revealed that the promoter region exhibited an obvious reduction of nucleotide diversity in both *indica* and *japonica* relative to that in the wild rice (Table S3), potentially indicating that this region might be targeted by selection. However, the diversity ratio in the promoter region of *Ghd8* between *japonica* and wild rice is slightly higher than that between *indica* and wild rice. The neutrality test revealed that an approximately 25-kb genome region surrounding *Ghd8* was subjected to selection, with a highly positive Tajima’s D value (average = 2.2) in the *japonica* group. By contrast, no significant Tajima’s D (average = −0.01) was detected in the same region of *indica* (Fig. S8). The significant and positive Tajima’s D value suggests that selection occurred in the *Ghd8* region, thereby causing prevalent haplotypes such as Pro13 and *Ghd8-1* in the *japonica* group growing in temperate areas.

## Discussion

Previous studies have shown that several variants occur in the coding region of the transcription factor *Ghd8* that alter the function on flowering time or heading date^8,13,23^. Variations in the expression levels of several flowering time genes due to cis-regulatory polymorphisms have been reported in maize and *Arabidopsis*^31,32^. In our study, we demonstrate that naturally occurring variants in the promoter region of *Ghd8* have effects on flowering time and cold tolerance in rice, which are two adaptive traits important for crop adaption in diverse environments.

One of the findings in the present study is that the functions of *Ghd8* are largely dependent on its transcription, with the upregulation of *Ghd8* not only delaying flowering time (Figs 2, 3) but also improving cold tolerance (Fig. 6). *Ghd8* was identified as a major locus affecting flowering with the dual function: inhibit flowering under LD conditions and promote flowering under SD conditions^23,26^. In the current study, this dual function of *Ghd8* on flowering was also found in the transgenic lines by regulating *Ehd1* and *Hd3a/RFT1* (Fig. 2, Fig. S3). Intriguingly, a high expression level of *Ghd8* enhanced the inhibition of *Ghd8* on flowering time by suppressing the expression of the downstream genes *Ehd1*-*Hd3a*/*RFT1* under both LD and SD conditions (Fig. S3). We further identified the variant T1279G that occurs in the promoter region of *Ghd8*, regulating *Ghd8* transcription, plays an important role in flowering time and cold tolerance in rice (Figs 4, 6). Several lines of evidence suggest that T1279G in the cis-regulatory region of *Ghd8* is highly associated with its transcription and contributes to the variations in flowering time and cold tolerance in rice. First, the promoter variations of *Ghd8* were clustered into two main haplotypes, which corresponded well to the subspecies *indica* and *japonica*. The haplotype *japonica* (e.g., Pro13) displayed a high expression of *Ghd8* relative to the haplotype *indica* (e.g., Pro3). However, we observed that the transgenic lines with a truncated Pro3 promoter (e.g., Pro3-1161) displayed an unexpected higher expression level of *Ghd8* than those with the Pro13 promoter in rice and *Arabidopsis*, suggesting there is a negative cis-regulatory element in Pro13 repressing the expression of *Ghd8*. Importantly, the elevated expression level of *Ghd8* in rice transgenic lines delayed the flowering time and increased the tolerance to low temperatures (Figs 2, 3, 6). Second, *GUS* transgenic lines revealed that the promoter lacking the negative cis-regulatory element within the distal region (−1285 to −1161 bp) of *Ghd8* induced the gene expression (Fig. 4, Fig. S4). This note was also supported by the transient assays using various truncated promoters in rice protoplasts. Third, site-mutated experiments provided a strong support for T1279G as a causal variant in the negative cis-regulatory element that regulates *Ghd8* expression.

Notably, the variant T1279G is located on the potential GA-responsive motif that might contribute to a different response to GA treatment. The 1279 T mutant revealed significantly increased expression compared to the 1279 G variant after the application of GA (Fig. 4d, Fig. S7). It has been reported that the pyrimidine box CCTTTT and the other two motifs, the TAACAAA box and TATCCAC box, together are required for a GA response in transiently transformed barley aleurone layers^30,33–35^. Damage to either of them caused a reduction of promoter expression in GA-treated aleurone layers, but did not completely abolish GA induction. Consistent with *Ghd8* being induced by GA, we found that a TATCCAC box (at −644 bp) and a TAACAAA box (at −572 bp) exist in the *Ghd8* promoter. Together with the CCTTTT motif at −1279 bp, they likely form a GA-responsive complex, which could play a role in regulating the *Ghd8* expression in response to GA. Furthermore, the *japonica* cultivars carrying the 1279 T variant had a higher expression level of *Ghd8* compared to the *indica* cultivars that harbored the 1279 G variant (Fig. 6f). Therefore, we suggest that low temperatures might activate the GA-related genes that recognize the pyrimidine motif at the *Ghd8* promoter to induce the expression of *Ghd8*, although this requires further experimental verification.

The other finding in our investigation is that regulation of *Ghd8* is necessary for rice adaptability. Our results indicate that the divergent variants in the promoter region and coding region of *Ghd8* produce functional diversity in flowering time and cold tolerance, which driving rice adaption to specific environments. Haplotype analyses in rice accessions have revealed that *Ghd8* has two major haplotypes showing some *indica-japonica* specificity. One major haplotype, GHD8-1, with function at a high frequency (70.1%) exists in the *japonica* rice grown in temperate areas or northern latitudes (Fig. S9). The other haplotypes, such as GHD8-5 and GHD8-8 with nonfunctional, occur in 21% and 16% of *indica* cultivars, respectively, that are widely distributed throughout the subtropical regions. Different geographical distributions of these haplotypes potentially indicate that they might have some ability to adapt to particular environments. A contrasting pattern of distribution was reported for the flowering gene *Ghd7*; the strong function alleles of *Ghd7* tend to be found in the southern parts of Asia, and weaker or nonfunctional alleles appear more frequently in the northern region^13^. It is believed that the northward expansion of rice into the long-day environments is largely facilitated by early flowering, reduced photoperiod sensitivity and tolerance to low temperature. In terms of cold tolerance, it is an ecologically significant trait for the adaptation of plants to cold climates. However, molecular evidence supporting such a claim is very limited. We found that all of the *japonica* cultivars from high latitudes or northern regions carry the promoter haplotype with the 1279 T variant accompanied by the *Ghd8-1* allele, which has a function on flowering time^23^. It is particularly interesting that the *japonica* rice with the 1279 T variant is strongly associated with a high expression of *Ghd8-1*, increasing cold tolerance. In parallel, we observed that the *Ghd8-5* and *Ghd8-8* alleles experienced a loss of function and appear more frequently in *indica* cultivars, which might have occurred later during rice domestication or breeding selection, since they were not detected in the wild progenitors (Fig. S1). Regarding the alleles of *Ghd8* with a strong function on flowering time^13,23^, a lower expression of the functional alleles is preferable in rice if grown at lower latitudes, otherwise a high expression would result in a seriously delayed flowering time that might be harmful to productivity in southern regions. Thus, weak alleles with a moderate effect on photo-sensitivity and a strong effect on cold tolerance would facilitate expanding rice growing to the northern regions. Consistent with this proposal, most *indica* accessions grown in southern regions carry the 1279 G variant of *Ghd8* and confer a lower expression level compared to the *japonica* rice. The distribution of rice is closely correlated with latitudes might result from the joint functional effects of regulatory and coding variants in the *indica-japonica* breeding history. Our study provides a novel view of the functional variations in the promoter region of *Ghd8*, offering a powerful means to fine-tune gene expression for rice adaptability in various environments.

It has been reported that cis-regulatory element modifications are tend to be the targets that undergo selection during plant domestication^36–39^. We propose that *Ghd8* is a likely target of selection during rice domestication based on three lines of evidence. First, the promoter region exhibits an obvious reduction of nucleotide diversity in cultivated rice relative to wild rice (Table S3). Second, genetic divergence of the *Ghd8* promoter is found in the cultivated rice, and a high level of diversity is maintained at the *Ghd8* locus in both subspecies. Third, a significant positive Tajima’s D value is detected in the 25-kb genome fragment harboring *Ghd8* in the *japonica* cultivars and indicates a strong haplotype structure appearing in the subspecies *japonica* (Fig. S8). This reflects that some certain GHD8 protein types are always linked to a specific promoter with the divergent T1279G. As mentioned above, this SNP variant is most likely to be responsible for the expression change. Our results provide a new example supporting the theory that cis-regulatory variations underwent selection for local adaptation during rice domestication.

In conclusion, we reveal that cis-regulatory variation in the *Ghd8* promoter, along with the coding region function, is critical for rice to adapt to different regions. The T1279G variant, located on a negative cis-regulatory region of *Ghd8*, causes a high expression level of *Ghd8* in *japonica* rice and leads to a change in cold tolerance, thus contributing significantly to the ecological adaptation of rice varieties at high latitudes or in northern regions. These findings provide insights into the cis-regulatory variant that functionally affects gene expression associated with adaptive traits in rice.

## Materials and Methods

### Plant materials and growth conditions

A rice mini-core collection comprised of 198 rice accessions was used in the nucleotide diversity analysis^40,41^. For function analysis of *Ghd8* regulating flowering time, the T-DNA mutant PFG_3A-18183 in cv. Hwayoung background was obtained from POSTECH RISD (http://www.postech.ac.kr/life/pfg/risd/). The homozygous T-DNA insertion mutants were confirmed by using border primers on the rice genome and the vector T-DNA (Table S4). The homozygous mutant lines, along with the negative plants were grown in natural LD conditions to score flowering time. We also obtained the *Arabidopsis* mutant SALK_025666 *hap3b* from the Nottingham Arabidopsis Stock Centre for function analysis of *Ghd8*.

The overexpression line of *Ghd8* was generated by using the plasmid pCAMBIA-1301, with the *Ghd8* coding region from the *japonica* cultivar Nipponbare driven by a plant *Ubiquitin* promoter. Artificial microRNA (amiRNA) lines (named amiRNA-*Ghd8*) were generated, as previously described^42^. Using the WMD3 tool (http://wmd3.weigelworld.org), we designed 21-mer small RNA carrying the mismatch sites at *Ghd8* to create amiRNA-*Ghd8* that specifically silenced the gene expression. The mutant and transgenic lines were grown in growth chambers under LD conditions (14.5 h light: 9.5 h dark). Three transgenic lines for each construct were analyzed for the phenotype of interest. The flowering time and leaf number was scored in *Arabidopsis* mutant, as previously described^43^.

### Gene expression analyses

Rice leaves were harvested from the 35-day-old seedlings for expression analysis. Rice plants were grown under both natural LD conditions (14.5 h light: 9.5 h dark) and SD conditions (9 h light: 15 h dark). Total RNA was extracted from leaf tissues using TRIzol and was treated with DNase I following the TRIzol reagent protocol (Invitrogen). cDNA (20 µL) was synthesized from 1 µg of RNA using SuperScriptII Reverse Transcriptase (Invitrogen). One microliter of cDNA was used for real-time PCR with SYBR Green PCR master mix (Applied Biosystems). The data were collected using an Applied Biosystems 7500 Real-time PCR System. All expression levels were normalized to that of the *ubiquitin* gene using the relative quantification method^44^.

### Transgenic analysis for functional variations of *Ghd8*

To compare the transcription levels caused by variation in the *Ghd8* promoter region, two constructs with a binary vector pCAMBIA1301 were generated and transformed into the recipient cv. Zhenshan97B (ZS97). The first construct, named as NN, contained a 3.8-kb fragment from cv. Nipponbare, including 1469-bp of promoter region (Pro13) upstream of the ATG initiation codon of *Ghd8*, 893-bp of coding region, and approximately 1400-bp of the 3′ UTR^23^. The second construct, designated as ZN, was created by using a promoter fragment (from −1186 bp to ATG) from ZS97 driving the identical coding region of *Ghd8* from Nipponbare. The ZN and NN constructs were also introduced into the *Arabidopsis hap3b* mutant using the floral dipping method^45^.

To study whether the *Ghd8* promoter variants altered the expression of *Ghd8*, two main types of promoter: Pro3 (named Pro3-1469) and Pro13 (named Pro13-1469), were respectively isolated from ZS97 and Nipponbare by PCR and inserted into the DX2181 vector that carries a *GUS* (β-glucuronidase) reporter gene^46^. These two constructs were transformed into the *japonica* variety Hejiang19 (HJ19) by the *agrobacterium*-mediated transformation^47^. In addition, four truncated promoters from the above Pro3 and Pro13 were amplified by PCR and inserted into the DX2181 vector as well. The truncated *Ghd8* promoter constructs were transformed into cv. HJ19 or Zhonghua 11 (ZH11) by the *agrobacterium*-mediated method.

### Luciferase transient expression assay in rice protoplasts

To construct the reporter Pro3-1161::LUC, Pro3-1279G::LUC and Pro3-1279T::LUC, the segments of *Ghd8* promoter (Pro3-1161, Pro3-1279G, Pro3-1279T) were amplified by PCR and cloned into the pGreen II 0800-LUC vector using the *Hind* III and *BamH* I restriction sites^48^. Rice protoplast isolation and transfection were based on the method described in a published protocol^49^. For cotransfection assays, 5 µg of construct were used for each polyethylene glycol transfection. After 12 h of transient transformation, protoplasts were incubated for 2 h with an addition of the gibberellic acid (GA3) at a final concentration of 5 μM at 28 °C under dark conditions for luciferase (LUC) activity measurement. The Renilla luciferase gene (rLUC), driven by the 35 S promoter of Cauliflower mosaic virus (CaMV), was used as an internal control. Firefly and Renilla luciferase activity were quantified using a Dual-Luciferase Reporter Assay System (Promega; No. E1910), according to the manufacturer’s instructions, and chemiluminescence was measured using a TECAN Infinite M200 microplate. Three replicates were performed.

### DNA sequence analysis

The promoter region of *Ghd8* was amplified by using the ExTaq and GC buffer I (Takara) from genomic DNA of the 198 rice accessions. The purified PCR products were used as templates and sequenced using the Big Dye Terminator v3.1 Cycle sequencing kit (Applied Biosystems). All sequences were assembled using Sequencher v4.5 software and aligned against the Nipponbare sequence of *Ghd8* (LOC_Os08g07740.1) downloaded from the website http://rice.plantbiology.msu.edu/. A phylogenetic tree was constructed for various *Ghd8* alleles using MEGA software (https://www.megasoftware.net/). The number beside the branches obtained by a bootstrapping approach represents a measure of support for the node, where 100 represents maximal support. Diversity analysis and Tajima’s D analysis were performed with a 2-kb window size based on a previous method^50^ for the 100-kb region surrounding *Ghd8* in 156 *japonica* and 295 *indica* varieties, which were downloaded from the website http://ricevarmap.ncpgr.cn/. All primers used for PCR and quantitative real-time PCR are listed in Table S4.

### Cold tolerance experiment

To test cold stress tolerance, rice accessions, near-isogenic lines (NIL) of *Ghd8*, and the NN and ZN transgenic lines were used. Thirty-six germinated seeds for each sample were sown in 6 × 6 × 10 (cm) pots and placed in a 30 °C growth chamber for 7 days. They were then transferred into a 4 °C chamber for 18 h for the transgenic lines, or 24 h for the natural rice accessions or NILs. After the cold treatment, the seedlings were returned to a 30 °C growth chamber for recovery. The survival rate was determined after 6 days of recovery growth by counting the percentage of the total seedlings that were alive, as previously described^51^. The cold tolerance experiment was replicated three times.

## Supplementary information

Supplemental figures and tables

## Data Availability

All data generated or analysed during this study are included in this published article and its Supplementary Information files.

## References

[1] Takahashi Y, Teshima KM, Yokoi S, Innan H, Shimamoto K (2009). Variations in Hd1 proteins, Hd3a promoters, and Ehd1 expression levels contribute to diversity of flowering time in cultivated rice. Proc Natl Acad Sci USA.

[2] Koo BH (2013). Natural variation in OsPRR37 regulates heading date and contributes to rice cultivation at a wide range of latitudes. Mol Plant.

[3] Xue W (2008). Natural variation in Ghd7 is an important regulator of heading date and yield potential in rice. Nat Genet.

[4] Matsubara K (2012). Natural variation in Hd17, a homolog of Arabidopsis ELF3 that is involved in rice photoperiodic flowering. Plant Cell Physiol.

[5] Saito H (2012). Ef7 encodes an ELF3-like protein and promotes rice flowering by negatively regulating the floral repressor gene Ghd7 under both short- and long-day conditions. Plant Cell Physiol.

[6] Zhao J (2012). OsELF3-1, an ortholog of Arabidopsis early flowering 3, regulates rice circadian rhythm and photoperiodic flowering. PLoS One.

[7] Yang Y, Peng Q, Chen GX, Li XH, Wu CY (2013). OsELF3 is involved in circadian clock regulation for promoting flowering under long-day conditions in rice. Mol Plant.

[8] Wei X (2010). DTH8 suppresses flowering in rice, influencing plant height and yield potential simultaneously. Plant Physiol.

[9] Dai X (2012). LHD1, an allele of DTH8/Ghd8, controls late heading date in common wild rice (Oryza rufipogon). J Integr Plant Biol.

[10] Zhao J (2015). Genetic interactions between diverged alleles of Early heading date 1 (Ehd1) and Heading date 3a (Hd3a)/ RICE FLOWERING LOCUS T1 (RFT1) control differential heading and contribute to regional adaptation in rice (Oryza sativa). New Phytol.

[11] Gao H (2013). Ehd4 encodes a novel and Oryza-genus-specific regulator of photoperiodic flowering in rice. PLoS Genet.

[12] Wu W (2013). Association of functional nucleotide polymorphisms at DTH2 with the northward expansion of rice cultivation in Asia. Proc Natl Acad Sci USA.

[13] Zhang J (2015). Combinations of the Ghd7, Ghd8 and Hd1 genes largely define the ecogeographical adaptation and yield potential of cultivated rice. New Phytol.

[14] Matsubara K (2011). Ehd3, encoding a plant homeodomain finger-containing protein, is a critical promoter of rice flowering. Plant J.

[15] Shibaya T (2016). Hd18, encoding histone acetylase related to Arabidopsis FLOWERING LOCUS D, is Involved in the control of flowering time in rice. Plant Cell Physiol.

[16] Hayama R, Yokoi S, Tamaki S, Yano M, Shimamoto K (2003). Adaptation of photoperiodic control pathways produces short-day flowering in rice. Nature.

[17] Komiya R, Ikegami A, Tamaki S, Yokoi S, Shimamoto K (2008). Hd3a and RFT1 are essential for flowering in rice. Development.

[18] Dolfini D, Mantovani R (2013). YB-1 (YBX1) does not bind to Y/CCAAT boxes *in vivo*. Oncogene.

[19] Laloum T, De Mita S, Gamas P, Baudin M, Niebel A (2013). CCAAT-box binding transcription factors in plants: Y so many?. Trends Plant Sci.

[20] Petroni K (2012). The promiscuous life of plant NUCLEAR FACTOR Y transcription factors. Plant Cell.

[21] Xu L (2014). Multiple NUCLEAR FACTOR Y transcription factors respond to abiotic stress in Brassica napus L. PLoS One.

[22] Mantovani R (1999). The molecular biology of the CCAAT-binding factor NF-Y. Gene.

[23] Yan WH (2011). A major QTL, Ghd8, plays pleiotropic roles in regulating grain productivity, plant height, and heading date in rice. Mol Plant.

[24] Yano M (2000). Hd1, a major photoperiod sensitivity quantitative trait locus in rice, is closely related to the Arabidopsis flowering time gene CONSTANS. Plant Cell.

[25] Goretti D (2017). Transcriptional and post-transcriptional mechanisms limit heading date 1 (Hd1) function to adapt rice to high latitudes. PLoS Genet.

[26] Du A (2017). The DTH8-Hd1 module mediates day-length-dependent regulation of rice flowering. Mol Plant.

[27] Ito Y (2006). Functional analysis of rice DREB1/CBF-type transcription factors involved in cold-responsive gene expression in transgenic rice. Plant Cell Physiol.

[28] Ohtsu K (2002). ABA-independent expression of rice alternative oxidase genes under environmental stresses. Plant Biotechnology.

[29] Higo K, Ugawa Y, Iwamoto M, Korenaga T (1999). Plant cis-acting regulatory DNA elements (PLACE) database: 1999. Nucleic Acids Res.

[30] Rogers JC, Rogers SW (1992). Definition and functional implications of gibberellin and abscisic acid cis-acting hormone response complexes. The Plant Cell.

[31] Rosas U (2014). Variation in Arabidopsis flowering time associated with cis-regulatory variation in CONSTANS. Nat Commun.

[32] Guo L (2018). Stepwise cis-regulatory changes in ZCN8 contribute to maize flowering-time adaptation. Curr Biol.

[33] Gubler F, Jacobsen JV (1992). Gibberellin-responsive elements in the promoter of a barley high-pI alpha-amylase gene. Plant Cell.

[34] Rogers JC, Lanahan MB, Rogers SW (1994). The cis-acting gibberellin response complex in high pI alpha-amylase gene promoters. Requirement of a coupling element for high-level transcription. Plant Physiol.

[35] Skriver K, Olsen FL, Rogers JC, Mundy J (1991). Cis-acting DNA elements responsive to gibberellin and its antagonist abscisic acid. Proc Natl Acad Sci USA.

[36] Li Y (2011). Natural variation in GS5 plays an important role in regulating grain size and yield in rice. Nat Genet.

[37] Zhao B (2018). Core cis-element variation confers subgenome-biased expression of a transcription factor that functions in cotton fiber elongation. New Phytol.

[38] Wang S (2015). The OsSPL16-GW7 regulatory module determines grain shape and simultaneously improves rice yield and grain quality. Nat Genet.

[39] Cong B, Liu J, Tanksley SD (2002). Natural alleles at a tomato fruit size quantitative trait locus differ by heterochronic regulatory mutations. Proceedings of the National Academy of Sciences.

[40] Ding Z, Wang C, Chen S, Yu S (2011). Diversity and selective sweep in the OsAMT1;1 genomic region of rice. BMC Evol Biol.

[41] Wang C, Chen S, Yu S (2011). Functional markers developed from multiple loci in GS3 for fine marker-assisted selection of grain length in rice. Theor Appl Genet.

[42] Schwab R, Ossowski S, Riester M, Warthmann N, Weigel D (2006). Highly specific gene silencing by artificial microRNAs in Arabidopsis. Plant Cell.

[43] Cai XN (2007). A putative CCAAT-binding transcription factor is a regulator of flowering timing in Arabidopsis. Plant Physiology.

[44] Livak KJ, Schmittgen TD (2001). Analysis of relative gene expression data using real-time quantitative PCR and the 2(-Delta Delta C(T)) Method. Methods.

[45] Clough SJ, Bent AF (1998). Floral dip: a simplified method for Agrobacterium-mediated transformation of Arabidopsis thaliana. Plant J.

[46] Ye R, Zhou F, Lin Y (2012). Two novel positive cis-regulatory elements involved in green tissue-specific promoter activity in rice (Oryza sativa L ssp.). Plant Cell Rep.

[47] Nishimura A, Aichi I, Matsuoka M (2006). A protocol for Agrobacterium-mediated transformation in rice. Nature Protocols.

[48] Hellens RP (2005). Transient expression vectors for functional genomics, quantification of promoter activity and RNA silencing in plants. Plant Methods.

[49] Zhang Y (2011). A highly efficient rice green tissue protoplast system for transient gene expression and studying light/chloroplast-related processes. Plant Methods.

[50] Wang M (2014). The genome sequence of African rice (Oryza glaberrima) and evidence for independent domestication. Nat Genet.

[51] Ma Y (2015). COLD1 confers chilling tolerance in rice. Cell.

