# Supplementary material for: A key variant in the cis-regulatory element of flowering gene Ghd8 associated with cold tolerance in rice
Source: Sci Rep. 2019 Jul 3;9:9603. doi: 10.1038/s41598-019-45794-9 (PMC6610134; doi:10.1038/s41598-019-45794-9)
Supplement: Supplementary file 1 — Supplemental figures and tables [file 41598_2019_45794_MOESM1_ESM.pdf]

A key variant in the cis-regulatory element of flowering gene *Ghd8* associated with cold tolerance in rice

Peng Wang[1, 2, #], Yin Xiong[1, #], Rong Gong[1], Ying Yang[1,2], Kai Fan[1], Sibin Yu[1,*]

[1] National Key Laboratory of Crop Genetic Improvement, College of Plant Science and Technology, Huazhong Agricultural University, Wuhan 430070, China

[2] Current address: Department of Agronomy and Horticulture, University of Nebraska Lincoln, Lincoln, NE 68588-0660, USA

[#] Contribute equally to this study

*Author for correspondence: Sibin Yu, ysb@mail.hzau.edu.cn

Supplemental Figure and Table Legends

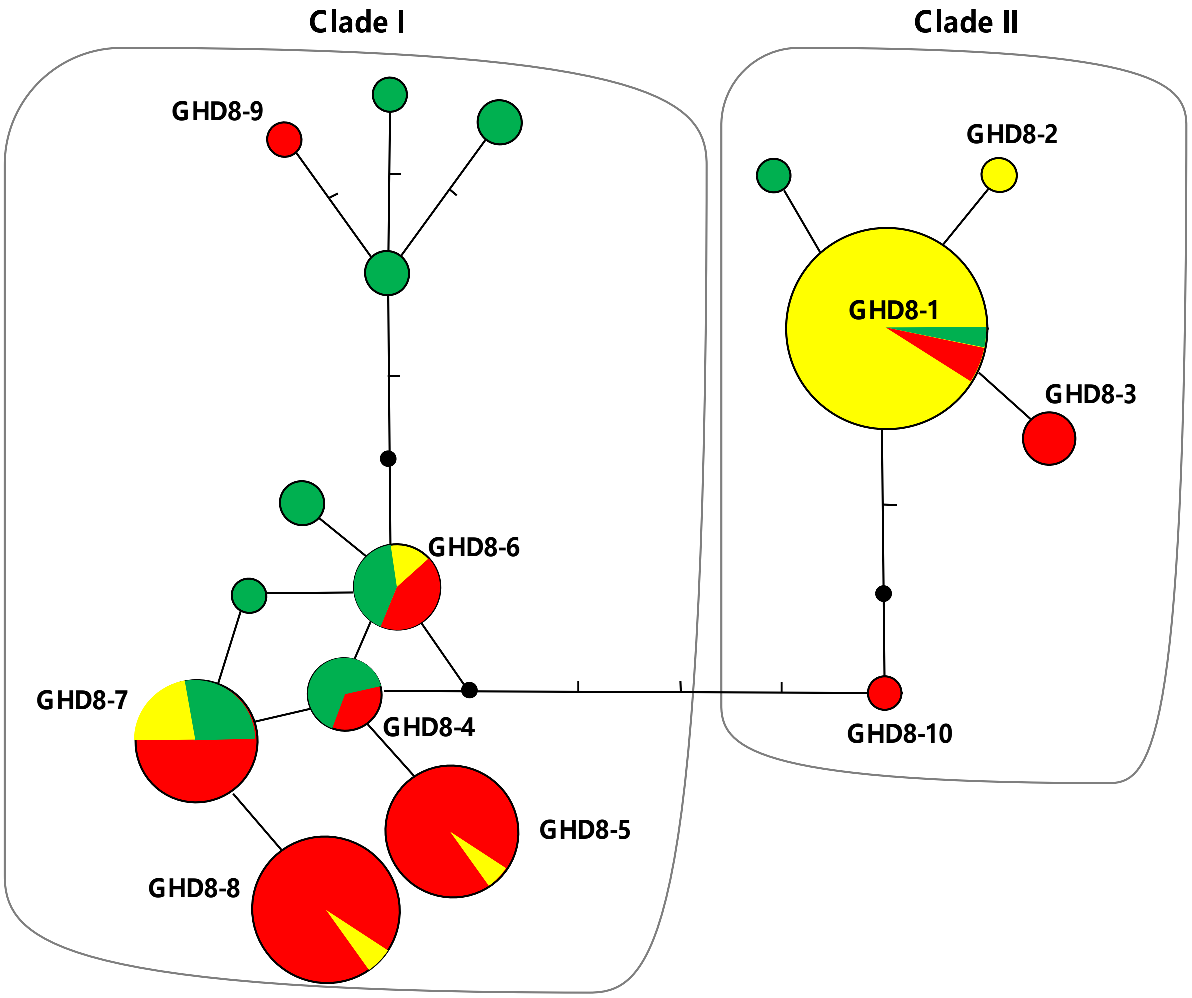

Fig. S1 Network analysis of GHD8 proteins in both cultivated rice and wild rice. Each pie represents a haplotype, and the size of the pie is proportional to the number of lines within a given haplotype. The yellow, red and green colors in the pie indicate *japonica*, *indica* and wild rice, respectively. The scale between each pair of pies indicates the number of mutation or variation differences between each two haplotypes.

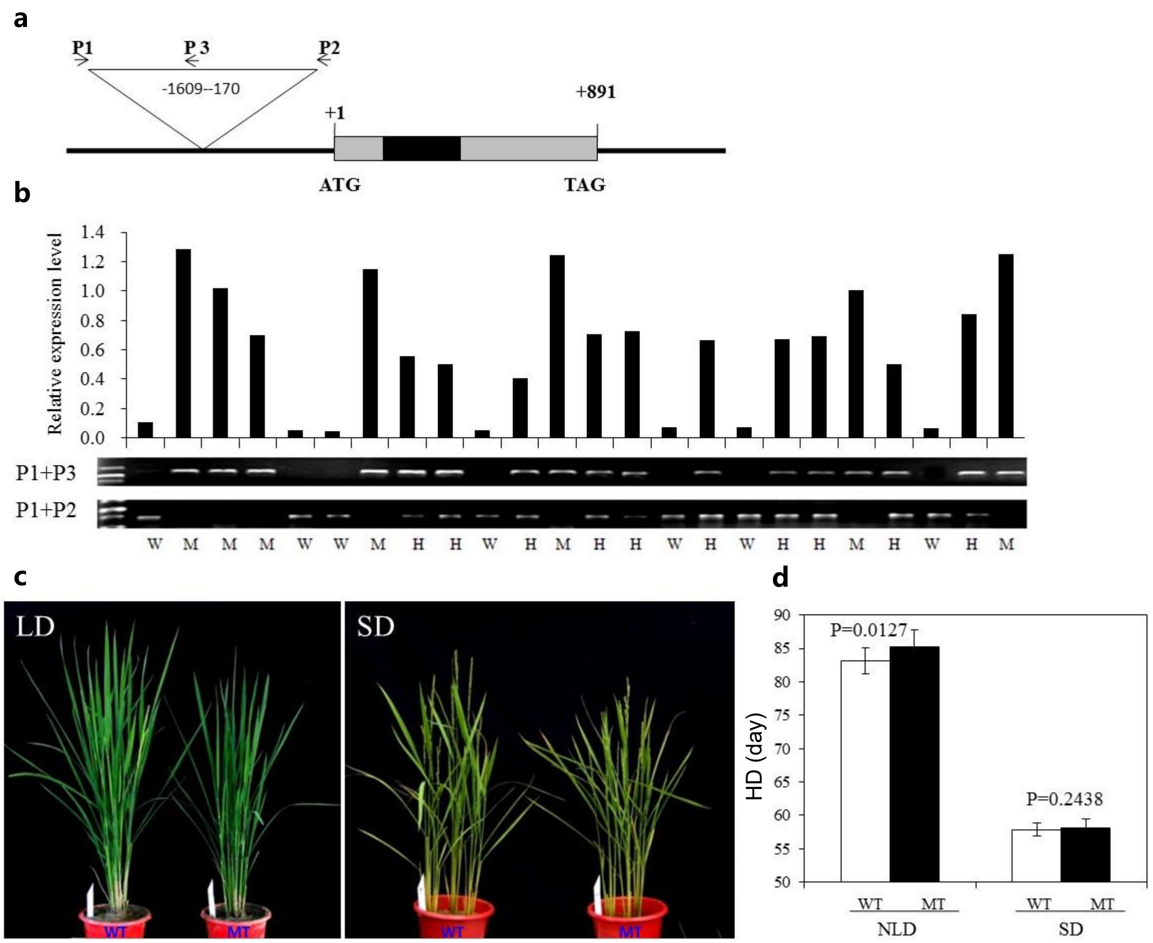

Fig. S2 Identification of a T-DNA insertion mutant in the promoter region of *Ghd8*. (a) The insertion site shown in the promoter of *Ghd8*. P1, P2 and P3 indicate the primers for checking the genotypes in a segregating population. (b) qRT-PCR analysis for the increased expression level of *Ghd8* in the T-DNA mutants. P1+P3 for insertion fragment, P1+P2 for fragment amplified from the promoter of *Ghd8*. W: wild type, M: homozygous mutant, H: heterozygous. (c) Performances of the wild type and homozygous mutant at the flowering stage in both long-day conditions (LD) and short-day conditions (SD). (d) Heading date (HD) of WT and MT (mutant). Student's *t*-test determines significant differences between WT and MT. Error bars indicate the mean ± SE (n = 15).

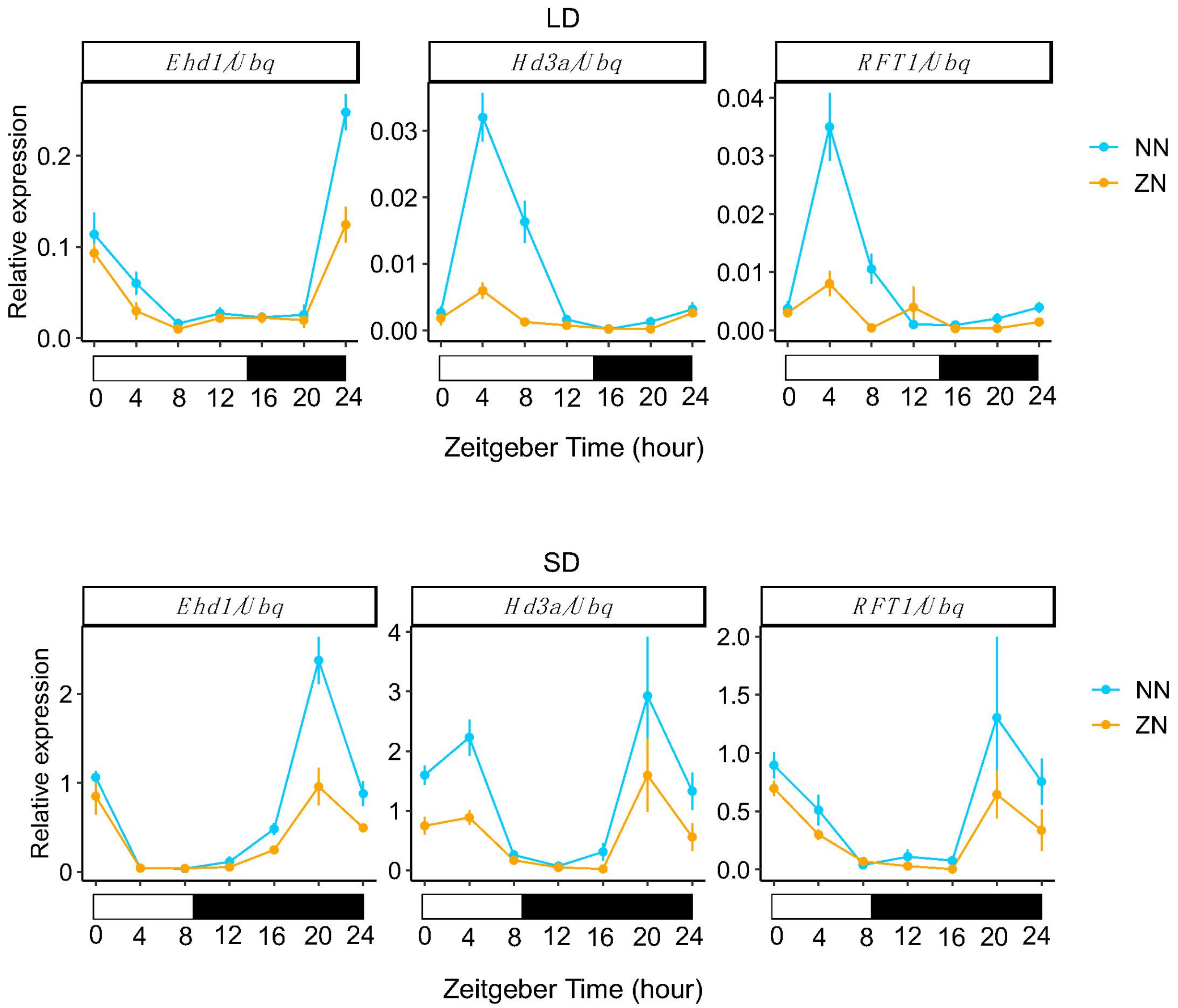

Fig. S3 Relative expression levels of downstream flowering genes *Ehd1*, *Hd3a*, and *RFT1* in the ZN and NN rice transgenic lines under LD and SD conditions. The white and black bars under the x-axis represent light and dark periods, respectively. The zeitgeber times on the axis are corresponding to the sampling times. Leaf samples were collected every 4 h from 35-day-old plants. Error bars indicate the mean ± SE with three replicates.

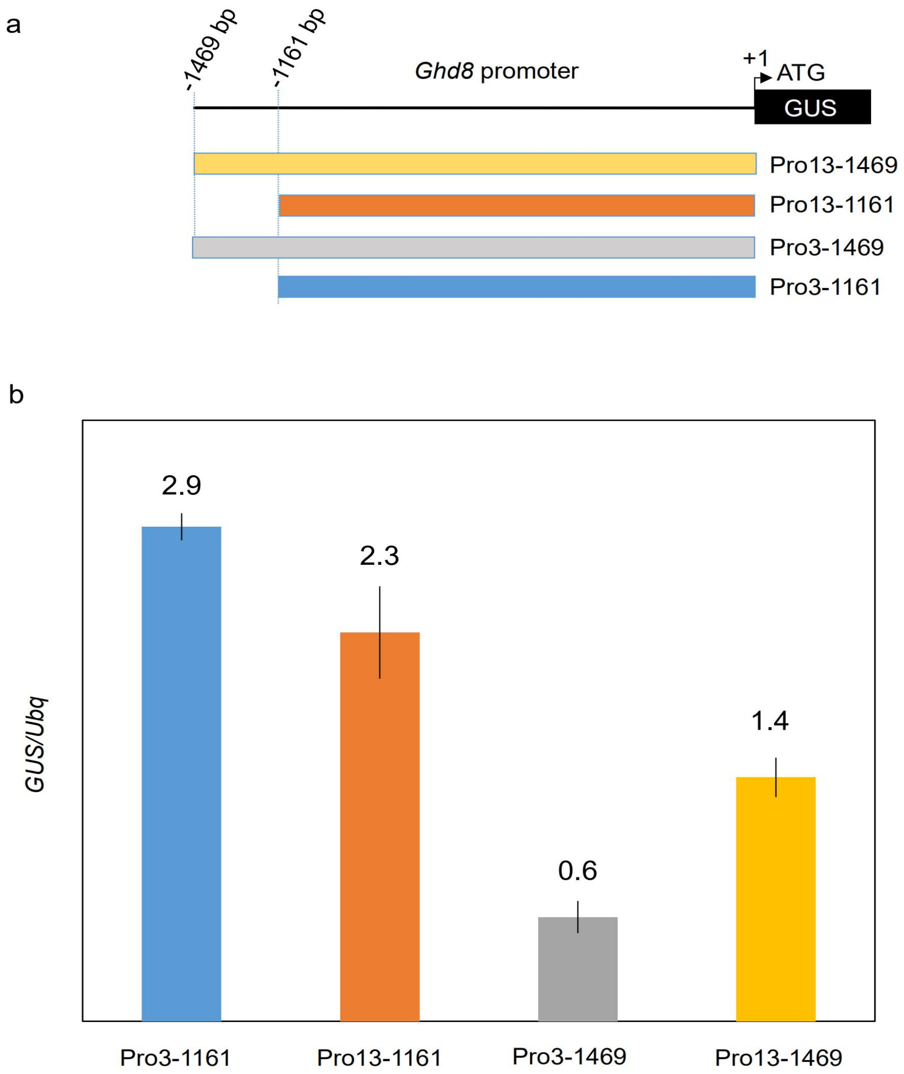

Fig. S4 Expression analyses using *GUS* reporter driven by different truncated *Ghd8* promoters in the transgenic plants of *japonica* recipient Zhonghua11. (a) Schematic illustration of the constructs used for the *GUS* gene reporter driven by a given promoter Pro3-1469, Pro13-1469, Pro3-1161 or Pro13-1161. (b) GUS expression levels in the young leaves of the transgenic plants in (a).

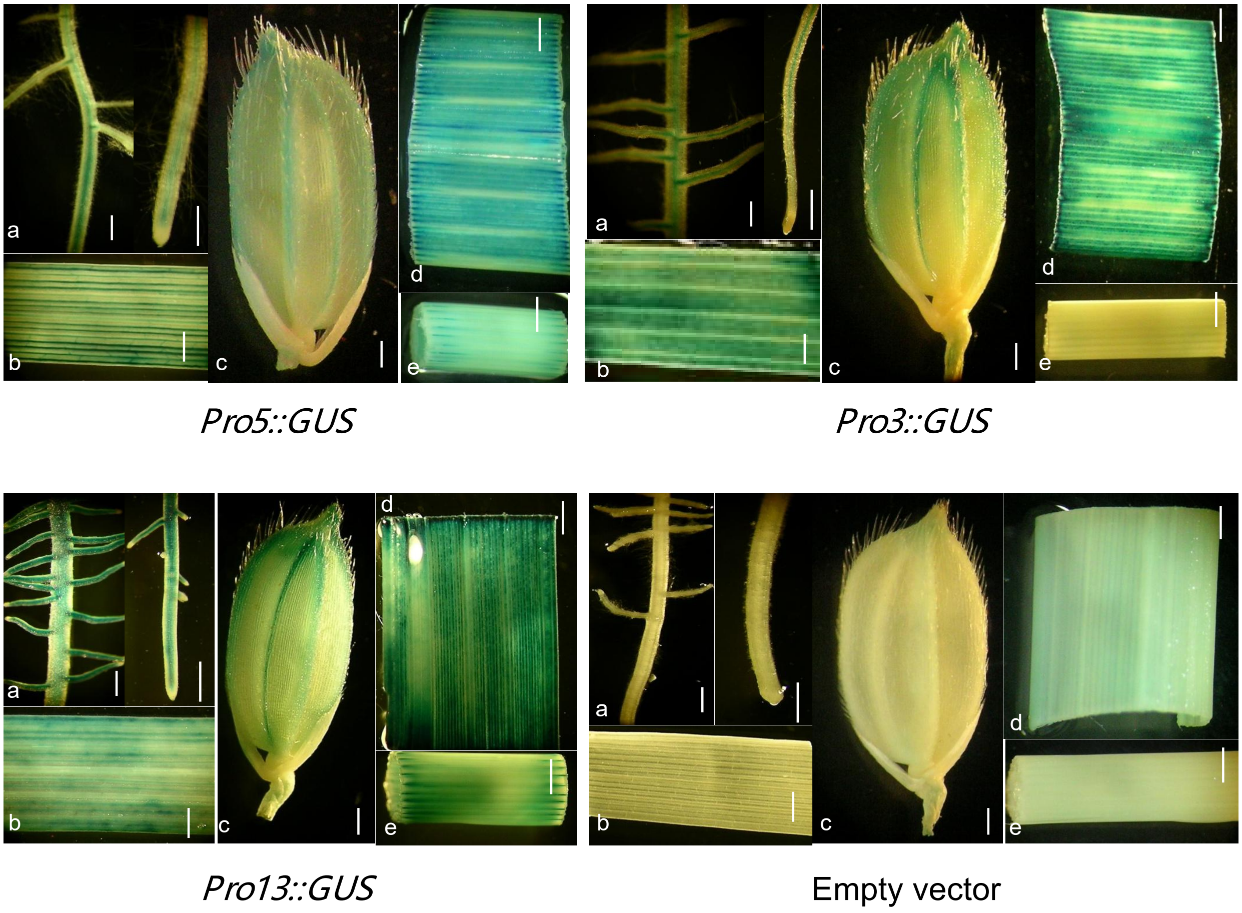

Fig. S5 GUS (β-glucuronidase) staining for transgenic plants of Pro5::*GUS*, Pro3::*GUS*, Pro13::*GUS* and empty vector in HJ19 background. The lateral roots (a left, bar: 1 mm), root tip (a right, bar: 1 mm), young leave (b, bar : 1 mm), seeds in the early filling stage (c, bar: 0.5 mm), flag leaves (d, bar: 2 mm), stem (e, bar: 0.15 mm). Photos were taken by Stereo Fluorescence Microscope Nikon SMZ800.

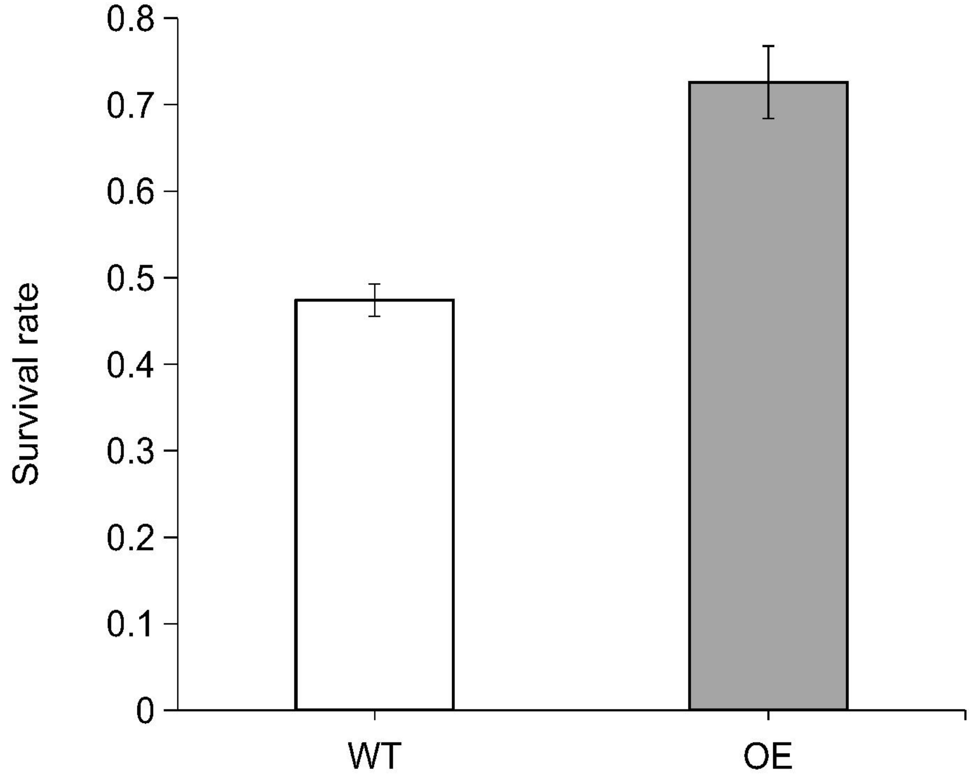

Fig. S6 Overexpression of *Ghd8* conferring better cold tolerance relative to wild type in rice. OE: overexpression lines. WT: wild type.

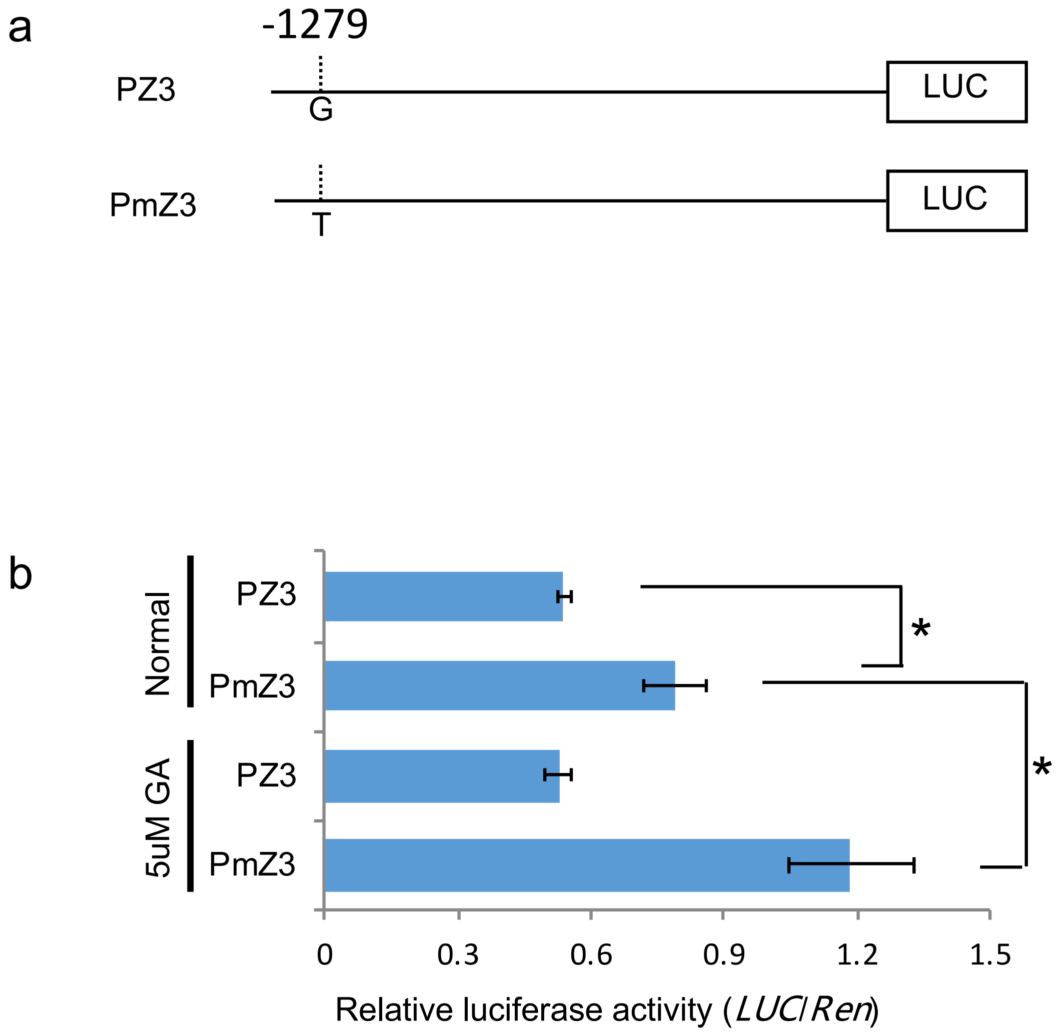

Fig. S7 Luciferase (LUC) transient expression assays for the variant T1279G in the *Ghd8* promoter, showing the variant 1279G replacement with 1279T increases the expression level of *Ghd8*. (a) Schematic diagram of the *LUC* constructs PmZ3 (Pro3-1279T) and PZ3 (Pro3-1279G), showing the T1279G position in the promoter of *Ghd8*. (b) The promoter PmZ3 significantly increases the response to GA treatment compared with PZ3. Asterisks indicate a significant difference at $p < 0.05$ by *t*-test.

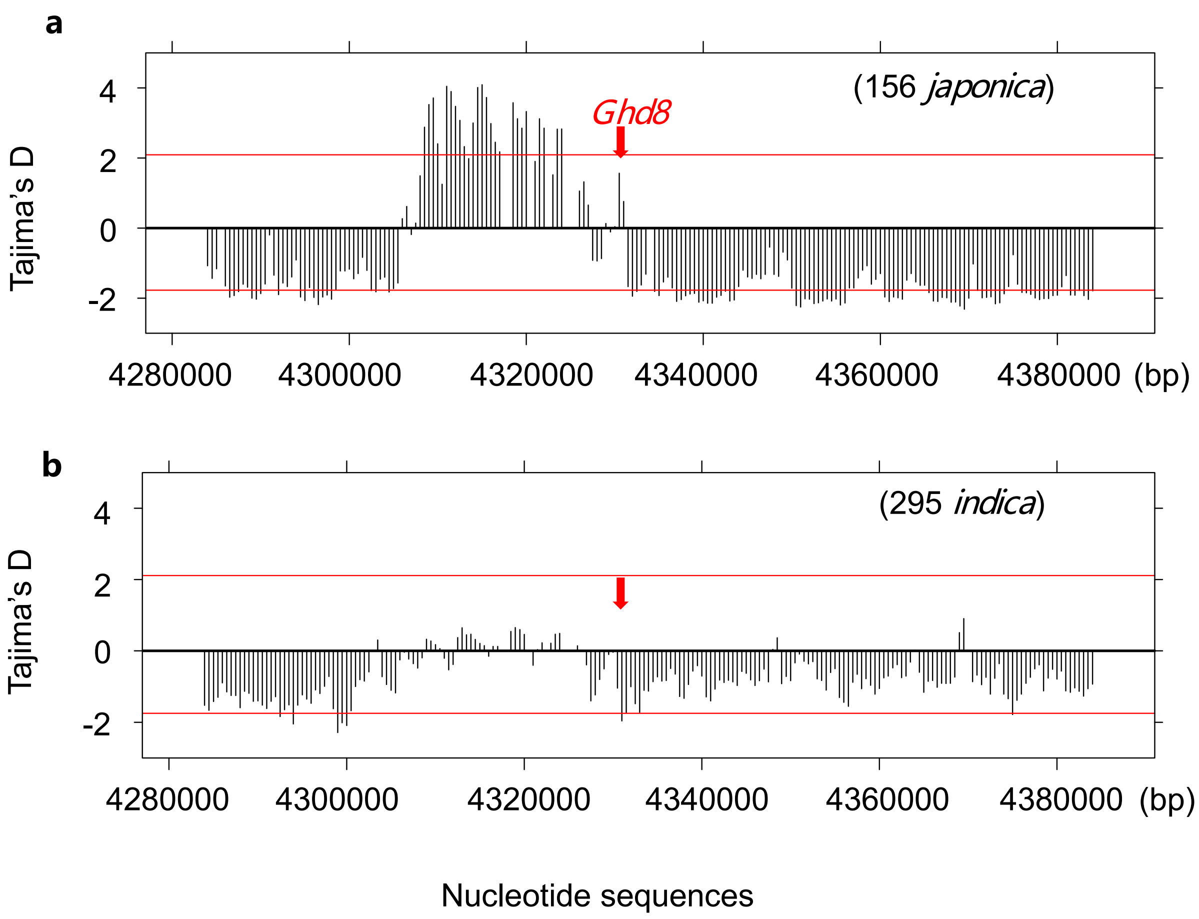

Fig. S8 Genome region encompassing *Ghd8* in subspecies *japonica* was under strong selection. Neutrality test for the 100-kb region surrounding the *Ghd8* gene shows the high positive Tajima's D values at the 25-kb fragment harboring *Ghd8* in *japonica* rice. The nucleotide sequences of 156 *japonica* cultivars (a) and 295 *indica* cultivars (b) were downloaded from http://ricevarmap.ncpgr.cn/.

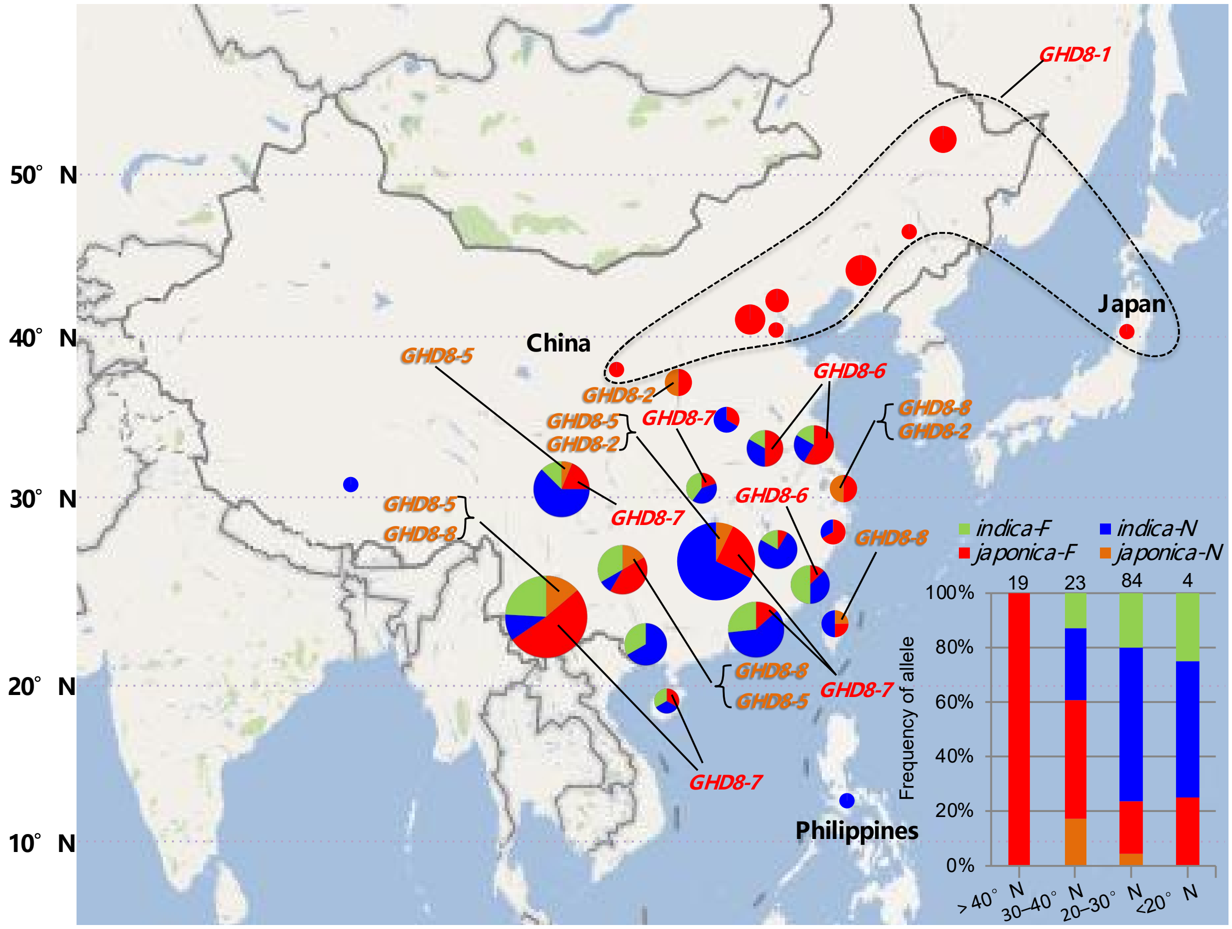

Fig. S9 Geographic distribution of the major protein haplotypes in the 198 rice accessions. The functional characters of the *Ghd8* alleles are shown. The green and blue in both pie and bar graphs indicate the functional (F) and nonfunctional (N) alleles in *indica*; the red and orange colors represent the functional and nonfunctional alleles in *japonica*, respectively. The size of pie represents the quantity of the accessions at the indicated latitudes. The stack bars on the right corner represent the percentage of accessions that carry different alleles in four regions from high to low latitudes.

Table S1 Information of 198 rice accessions used in this study

Planting ID	Variety name	Subspecies	Alleles	[a] Ghd8 function	Promoter haplotype
6W001	Hejiang	*japonica*	*Ghd8-1*	F	pro13
6W002	Heijing2	*japonica*	*Ghd8-1*	F	pro14
6W003	Laoguangtou83	*japonica*	*Ghd8-1*	F	pro13
6W004	Baimaodao	*japonica*	*Ghd8-1*	F	pro14
6W005	Erjiunan 1	*indica*	*Ghd8-5*	N	pro3
6W006	AnnongwanjingB	*japonica*	*Ghd8-1*	F	pro14
6W007	Chaoyangyihao B	*indica*	*Ghd8-8*	N	pro3
6W008	Aijiaonante	*indica*	*Ghd8-5*	N	pro3
6W009	L301B	*indica*	*Ghd8-5*	N	pro3
6W010	Guangluai 4	*indica*	*Ghd8-5*	N	pro3
6W011	Xiangaizao10	*indica*	*Ghd8-5*	N	pro3
6W012	Jinnante 43B	*indica*	*Ghd8-5*	N	pro3
6W013	Jing7623	*japonica*	*Ghd8-8*	N	pro3
6W014	Xiangzaoxian 7	*indica*	*Ghd8-5*	N	pro3
6W015	80B	*indica*	*Ghd8-5*	N	pro3
6W016	Guangluai 15	*indica*	*Ghd8-5*	N	pro3
6W017	ZhuzhenB	*indica*	*Ghd8-8*	N	pro3
6W018	Baoxie123B	*indica*	*Ghd8-8*	N	pro3
6W019	Zhenshan 97	*indica*	*Ghd8-5*	N	pro3
6W020	Heimangdao	*japonica*	*Ghd8-1*	F	pro13
6W021	Baoxie 7B	*indica*	*Ghd8-8*	N	pro3
6W022	JinnanteB	*indica*	*Ghd8-5*	N	pro17
6W023	Funingzipi	*japonica*	*Ghd8-1*	F	pro13
6W024	Zhenshan97B	*indica*	*Ghd8-5*	N	pro3
6W025	Gzhenshan97B	*indica*	*Ghd8-8*	N	pro3
6W026	Qingsiai16B	*indica*	*Ghd8-8*	N	pro3
6W027	LimingB	*japonica*	*Ghd8-1*	F	pro13
6W028	Nipponbare-1	*japonica*	*Ghd8-1*	F	pro13
6W029	Nanxiongzaoyou	*indica*	*Ghd8-3*	N	pro9
6W030	Jiangnongzao1	*indica*	*Ghd8-5*	N	pro3
6W031	Zaoshuxianghei	*indica*	*Ghd8-8*	N	pro3
6W032	IR661-1	*indica*	*Ghd8-8*	N	pro3
6W033	Nanjing 11	*indica*	*Ghd8-8*	N	pro3
6W034	Weiguo	*japonica*	*Ghd8-1*	F	pro13
6W035	Gui630	*indica*	*Ghd8-8*	N	pro3
6W036	Dianrui409B	*indica*	*Ghd8-8*	N	pro3
6W037	Liaojing287	*japonica*	*Ghd8-1*	F	pro13
6W038	Huhui628	*japonica*	*Ghd8-1*	F	pro13
6W039	IR65482-4-136-2-2	*indica*	*Ghd8-8*	N	pro3
6W040	JinghuB	*japonica*	*Ghd8-1*	F	pro13
6W041	88B	*indica*	*Ghd8-8*	N	pro3

6W042	Gu154	*indica*	*Ghd8-8*	N	pro3
6W043	Nipponbare	*japonica*	*Ghd8-1*	F	pro13
6W044	Ninghui21	*japonica*	*Ghd8-1*	F	pro13
6W045	Xianghui91269	*indica*	*Ghd8-8*	N	pro3
6W046	Longhuamaohu	*japonica*	*Ghd8-1*	F	pro12
6W047	Lucaihao	*indica*	*Ghd8-5*	N	pro3
6W048	Zhonglou1	*japonica*	*Ghd8-1*	F	pro13
6W049	Yezhongcanghua	*japonica*	*Ghd8-1*	F	pro13
6W050	Shuiyuan300	*japonica*	*Ghd8-1*	F	pro13
6W051	Shufeng101	*indica*	*Ghd8-3*	N	pro13
6W052	Chengduai 3	*indica*	*Ghd8-8*	N	pro3
6W053	XiangaiB	*indica*	*Ghd8-8*	N	pro3
6W054	Sankecun	*indica*	*Ghd8-7*	F	pro20
6W055	Gongju 73	*japonica*	*Ghd8-5*	N	pro3
6W056	Jiabala	*indica*	*Ghd8-5*	N	pro3
6W057	Taishannuo	*indica*	*Ghd8-5*	N	pro3
6W058	Guichao 2	*indica*	*Ghd8-5*	N	pro3
6W059	76-1	*japonica*	*Ghd8-1*	F	pro13
6W060	Xugunuo	*japonica*	*Ghd8-5*	N	pro3
6W061	Huke 3	*indica*	*Ghd8-5*	N	pro3
6W062	Taizhongzailai 1	*indica*	*Ghd8-8*	N	pro3
6W063	Teqingxuanhui	*indica*	*Ghd8-8*	N	pro3
6W064	Huangsiguizhan	*indica*	*Ghd8-5*	N	pro3
6W065	Aituogu 151	*indica*	*Ghd8-5*	N	pro3
6W066	Xiangwanxian 1	*indica*	*Ghd8-8*	N	pro3
6W067	Xiangwanxian3	*indica*	*Ghd8-5*	N	pro3
6W068	Taizhong65	*japonica*	*Ghd8-8*	N	pro3
6W069	Zaoshunonghu6	*japonica*	*Ghd8-1*	F	pro13
6W070	Jinyou 1	*indica*	*Ghd8-5*	N	pro3
6W071	Chengnongshuijing	*indica*	*Ghd8-8*	N	pro3
6W072	PeiC122	*japonica*	*Ghd8-7*	F	pro3
6W073	Minghui63	*indica*	*Ghd8-8*	N	pro3
6W074	Guihuahuang	*japonica*	*Ghd8-1*	F	pro13
6W075	Momi	*indica*	*Ghd8-5*	N	pro3
6W076	Xiushui115	*japonica*	*Ghd8-1*	F	pro13
6W077	Ergangai	*indica*	*Ghd8-5*	N	pro18
6W078	Sanbaili	*indica*	*Ghd8-3*	N	pro13
6W079	Jindao1	*japonica*	*Ghd8-1*	F	pro13
6W080	Xingguo	*japonica*	*Ghd8-1*	F	pro13
6W081	Dandongludao	*japonica*	*Ghd8-1*	F	pro13
6W082	Zhonghua8	*japonica*	*Ghd8-1*	F	pro13
6W083	Liusha 1	*indica*	*Ghd8-5*	N	pro3
6W084	Bawangbian 1	*japonica*	*Ghd8-7*	F	pro3
6W085	Aimakang	*indica*	*Ghd8-8*	N	pro3
6W086	Dongtingwanxian	*indica*	*Ghd8-7*	F	pro1

6W087	JWR221	*japonica*	*Ghd8-6*	F	pro3
6W088	Yangdao2	*indica*	*Ghd8-6*	F	pro4
6W089	93-11	*indica*	*Ghd8-6*	F	pro5
6W090	Zhengdao5	*japonica*	*Ghd8-1*	F	pro13
6W091	Zhenxian232	*indica*	*Ghd8-5*	N	pro3
6W092	Jing87-304	*japonica*	*Ghd8-1*	F	pro13
6W093	Sujing2	*japonica*	*Ghd8-1*	F	pro13
6W094	Hongwan1	*indica*	*Ghd8-7*	F	pro1
6W095	Liushizao	*indica*	*Ghd8-1*	F	pro13
6W096	Taidongludao	*japonica*	*Ghd8-1*	F	pro14
6W098	Muxiqiu	*japonica*	*Ghd8-2*	N	pro13
6W099	Taizhongxianxuan2	*indica*	*Ghd8-8*	N	pro3
6W100	Dangyu5	*japonica*	*Ghd8-6*	F	pro6
6W101	Binwan 3	*japonica*	*Ghd8-7*	F	pro3
6W102	Baoxuan 21	*indica*	*Ghd8-7*	F	pro17
6W103	Youmangzaojing	*japonica*	*Ghd8-1*	F	pro13
6W104	Maguzi	*japonica*	*Ghd8-1*	F	pro13
6W105	Qiuqianbai	*indica*	*Ghd8-8*	N	pro3
6W106	Menjiading2	*indica*	*Ghd8-5*	N	pro3
6W107	Aimi	*indica*	*Ghd8-5*	N	pro17
6W108	Haobayong1	*japonica*	*Ghd8-1*	F	pro15
6W109	Laohuzhong	*japonica*	*Ghd8-1*	F	pro13
6W110	Nantehao	*indica*	*Ghd8-5*	N	pro17
6W111	Wanlixian	*indica*	*Ghd8-5*	N	pro3
6W112	Esiniu	*indica*	*Ghd8-5*	N	pro3
6W113	Jiefangdao	*indica*	*Ghd8-5*	N	pro3
6W114	Cunsanli	*japonica*	*Ghd8-1*	F	pro14
6W115	Huangkezaonian	*japonica*	*Ghd8-1*	F	pro13
6W116	Nantiangangjiu	*japonica*	*Ghd8-1*	F	pro13
6W117	Benbanggu	*japonica*	*Ghd8-8*	N	pro3
6W118	Feidongtangdao	*japonica*	*Ghd8-1*	F	pro13
6W119	Hongqi5	*japonica*	*Ghd8-1*	F	pro8
6W120	Qitoubaigu	*japonica*	*Ghd8-5*	N	pro3
6W121	Hengxianliangchun	*indica*	*Ghd8-5*	N	pro3
6W122	Baikehualuo	*indica*	*Ghd8-5*	N	pro3
6W123	Muguanuo	*japonica*	*Ghd8-2*	N	pro13
6W124	Hanmadao	*indica*	*Ghd8-8*	N	pro3
6W125	Leihuozhan	*indica*	*Ghd8-5*	N	pro3
6W126	Heidu4	*indica*	*Ghd8-8*	N	pro3
6W127	Liuyenian	*indica*	*Ghd8-5*	N	pro3
6W128	Baikezaohe	*indica*	*Ghd8-5*	N	pro3
6W129	Gaoyangdiandao	*japonica*	*Ghd8-1*	F	pro13
6W130	Chikenuo	*indica*	*Ghd8-1*	F	pro13
6W131	Aihechi	*indica*	*Ghd8-7*	F	pro3
6W132	Haobuka	*japonica*	*Ghd8-1*	F	pro14

6W133	Haomake9(K)	*japonica*	*Ghd8-1*	F	pro14
6W134	Sanbangqishi	*japonica*	*Ghd8-1*	F	pro14
6W135	Shanjiugu	*japonica*	*Ghd8-1*	F	pro14
6W136	Fanhaopi	*indica*	*Ghd8-7*	F	pro19
6W137	Lixinjing	*japonica*	*Ghd8-1*	F	pro8
6W138	Xiangnuo	*japonica*	*Ghd8-1*	F	pro14
6W139	Niankenuo	*indica*	*Ghd8-1*	F	pro16
6W140	Yangkenuo	*indica*	*Ghd8-1*	F	pro13
6W141	Guantuibaihe1	*japonica*	*Ghd8-1*	F	pro10
6W142	Baigedao	*japonica*	*Ghd8-1*	F	pro13
6W143	Tieganwu	*japonica*	*Ghd8-1*	F	pro13
6W144	Sanlicun	*japonica*	*Ghd8-7*	F	pro3
6W145	Feienuo2	*indica*	*Ghd8-1*	F	pro14
6W146	Simiao	*indica*	*Ghd8-7*	F	pro3
6W147	Jixuenuo	*japonica*	*Ghd8-1*	F	pro13
6W148	Xisheng15	*japonica*	*Ghd8-1*	F	pro13
6W149	Xuanenchangtan	*indica*	*Ghd8-8*	N	pro3
6W150	Putaohuang	*japonica*	*Ghd8-1*	F	pro13
6W151	Xiangdao	*indica*	*Ghd8-8*	N	pro3
6W152	Meihuanuo	*indica*	*Ghd8-8*	N	pro3
6W153	Younian	*japonica*	*Ghd8-5*	N	pro3
6W154	Lamujia	*japonica*	*Ghd8-1*	F	pro15
6W155	Jinzhinuo	*indica*	*Ghd8-5*	N	pro3
6W156	Magunuo	*japonica*	*Ghd8-1*	F	pro13
6W157	Menjiagao 1	*japonica*	*Ghd8-7*	F	pro3
6W158	Xiaohonggu	*japonica*	*Ghd8-7*	F	pro3
6W159	Jinxibai	*indica*	*Ghd8-6*	F	pro7
6W160	Yanshuichi	*indica*	*Ghd8-7*	F	pro3
6W161	Guihezhan	*indica*	*Ghd8-7*	F	pro3
6W162	Jinbaoyin	*japonica*	*Ghd8-6*	F	pro7
6W163	Hongainuo	*indica*	*Ghd8-7*	F	pro3
6W164	Aizaizhan	*indica*	*Ghd8-7*	F	pro1
6W165	Dawannuo	*indica*	*Ghd8-7*	F	pro17
6W166	Baoerfu	*indica*	*Ghd8-6*	F	pro7
6W167	Zhongnong 4	*indica*	*Ghd8-5*	N	pro3
6W168	Laohongdao	*japonica*	*Ghd8-2*	N	pro10
6W169	Wuzidui	*japonica*	*Ghd8-1*	F	pro14
6W170	Xibainian	*japonica*	*Ghd8-5*	N	pro3
6W171	Dongtingwanxian	*indica*	*Ghd8-7*	F	pro3
6W172	Mibeiwanxian	*indica*	*Ghd8-7*	F	pro1
6W173	Babaili	*japonica*	*Ghd8-1*	F	pro14
6W174	Hongmisandan	*japonica*	*Ghd8-1*	F	pro13
6W175	Qiyuexian	*indica*	*Ghd8-7*	F	pro3
6W176	Qimei	*indica*	*Ghd8-7*	F	pro1
6W177	Yuyannuo	*japonica*	*Ghd8-1*	F	pro11

6W178	Zhegu	*japonica*	*Ghd8-8*	N	pro3
6W179	Maweizhan	*indica*	*Ghd8-5*	N	pro3
6W180	Mamagu	*indica*	*Ghd8-8*	N	pro3
6W181	Cungunuo	*indica*	*Ghd8-1*	F	pro10
6W182	Zinuo	*indica*	*Ghd8-5*	N	pro3
6W183	Lengshuigu2	*japonica*	*Ghd8-1*	F	pro14
6W184	Qingke	*indica*	*Ghd8-4*	F	pro8
6W185	Banjiemang	*japonica*	*Ghd8-1*	F	pro12
6W186	Beizinuo	*japonica*	*Ghd8-7*	F	pro3
6W187	Wuzuihonggu	*indica*	*Ghd8-7*	F	pro3
6W189	Nangaogu	*indica*	*Ghd8-7*	F	pro3
6W190	Hongkezhenuo	*japonica*	*Ghd8-1*	F	pro14
6W191	Mowanggunie	*japonica*	*Ghd8-7*	F	pro3
6W193	Haoluguangnian	*japonica*	*Ghd8-1*	F	pro14
6W194	Haocai	*indica*	*Ghd8-7*	F	pro3
6W195	Honggu	*indica*	*Ghd8-1*	F	pro14
6W196	Zimi	*japonica*	*Ghd8-5*	N	pro3
6W197	Qitougu	*indica*	*Ghd8-7*	F	pro2
6W198	Xianggu	*japonica*	*Ghd8-7*	F	pro3

[a] The coding region of *Ghd8* from 198 accessions were sequenced by using the specific primers described in Yan et al., 2011. F or N represents functional or nonfunctional alleles, respectively.

Table S2 Information of promoter and protein types of *Ghd8*

Promoter Haplotype [a]	No. of *indica*	No. of *japonica*	Representative accessions	Protein haplotype
Pro9	1		Nanxiongzaoyou	GHD8-3
Pro14	2	15	Babaili	GHD8-1
Pro16	1		Zhankenuo	GHD8-1
Pro15		2	Haobayong1	GHD8-1
Pro13	5	43	Nipponbare	GHD8-1, GHD8-2, GHD8-3
Pro12		2	Banjiemang	GHD8-1
Pro10	1	2	Guantuibaihe1	GHD8-1, GHD8-2
Pro11		1	Yuyannuo	GHD8-1
Pro1	5		Dongtingwanxian	GHD8-7
Pro17	5		Ergangai	GHD8-5, GHD8-7
Pro3	77	22	ZS97	GHD8-5, GHD8-6, GHD8-7, GHD8-8, GHD8-10
Pro2	1		Qitougu	GHD8-7
Pro18	1		JinnanteB	GHD8-5
Pro8	1	2	Qingke	GHD8-1, GHD8-4
Pro7	2	1	Jinxibai	GHD8-6
Pro5	1		93-11	GHD8-6
Pro6		1	Dangyu5hao	GHD8-6
Pro4	1		Yangdao2hao	GHD8-6
Pro20	1		Sankecun	GHD8-7
Pro19	1		Fanhaopi	GHD8-7

[a] Two clusters of the promoter haplotypes of *Ghd8* are indicated in Fig. S1. Several protein haplotypes linked with the promoter types are shown. The protein types in the red color font are highlighted as nonfunctional.

Table S3 Nucleotide diversity analysis in promoter, coding region and 3' UTR region of *Ghd8*

Parameters [a]	Categories	Entire region	Non-coding		Coding regions
			Promoter	3' region	
Length (bp)		4009	1843	1263	903
No. of Indels	*indica*	9	4	2	3
	japonica	13	3	6	4
	cultivated rice	13	3	6	4
	Wild rice	35	16	14	5
No. of SNP	*indica*	33	18	6	9
	japonica	54	19	25	10
	cultivated rice	62	21	31	10
	Wild rice	235	63	144	28
π/bp	*indica*	0.0022	0.0016	0.0056	0.0027
	japonica	0.0039	0.0030	0.0089	0.0045
	cultivated rice	0.005	0.0035	0.0109	0.0053
	Wild rice	0.0165	0.0078	0.0362	0.0053
θ/bp	*indica*	0.0025	0.0022	0.0057	0.0024
	japonica	0.0027	0.0022	0.0075	0.0025
	cultivated rice	0.0027	0.0021	0.0065	0.0021
	Wild rice	0.0151	0.0085	0.0293	0.0079
Tajima's D	*indica*	-0.4402	-0.7573	0.042	-0.0235
	japonica	1.4617	1.1494	0.5064	2.2785*
	cultivated rice	2.6867*	1.9324*	1.6643	3.6984**
	Wild rice	0.3497	-0.3036	0.9091	-1.1817
$\pi_{indica}/\pi_{rufipogon}$		0.1333	0.2051	0.1575	0.4528
$\pi_{japonica}/\pi_{rufipogon}$		0.2364	0.3846	0.2459	0.8491
$\pi_{Cultivar}/\pi_{rufipogon}$		0.3030	0.4487	0.3011	1.0000

[a] The diversity analysis was performed by using DnaSP 5.10 to across 30 wild rices (*O. rufipogon*), 46 *japonica* and 48 *indica* varieties. Cultivated rice *(Oryza sativa)* means the total of *japonica* and *indica* varieties used. ** P < 0.02, * P < 0.05.

Table S4 Primers used in this study

Name	Sequences (5'–3')	Remark
P1-Ghd8	GCAAGTGCCCATAGCTCTTC	identify insertion mutant
P3-Ghd8	TATATGCACTCGCAGATGCC	identify insertion mutant
P2-Ghd8	TTGGGGTTTCTACAGGACGTAAC	identify insertion mutant
Ubq-Forward	AACCAGCTGAGGCCCAAGA	qRT-PCR internal control
Ubq-Reverse	ACGATTGATTTAACCAGTCCATGA	qRT-PCR internal control
Hd3a-Forward	CTTCAACACCAAGGACTTCGC	qRT-PCR
Hd3a-Reverse	TAGTGAGCATGCAGCAGATCG	qRT-PCR
Ghd8-Forward	TCGCCGGACTCGTTGTCCAAC	qRT-PCR
Ghd8-Reverse	AGAGTAGGAAGAGCTATGGGC	qRT-PCR
Ehd1-Forward	GGATGCAAGGAAATCATGGA	qRT-PCR
Ehd1-Reverse	AATCCCATCGGAAATCTTGG	qRT-PCR
RFT1-Forward	TGACCTAGATTCAAAGTCTAATCCTT	qRT-PCR
RFT1-Reverse	TGCCGGCCATGTCAAATTAATAAC	qRT-PCR
DREB1a-Forward	ACCTGTACTACGCGAGCTTG	qRT-PCR
DREB1a-Reverse	TAGTAGCTCCAGAGTGGGAC	qRT-PCR
DREB1b-Forward	GATGGCGACGAAGAAGAAGA	qRT-PCR
DREB1b-Reverse	GAACCTGAACCCGTCGTC	qRT-PCR
AOX1b-Forward	TCAACCATTTCGCATCGG	qRT-PCR
AOX1b-Reverse	TCGCCCGTTGATGAATGA	qRT-PCR
H3b-Forward	AGTGTGTCTCCGAGTTCAT	identify hap3b
H3b-Reverse	TAGTCATAGCCCAGAGCAAA	identify hap3b
SOC-Forward	CTGAGGCATACTAAGGATCG	qRT-PCR
SOC-Reverse	GAACAAGGTAACCCAATGAA	qRT-PCR
FT-Forward	AGACGTTCTTGATCCGTTTA	qRT-PCR
FT-Reverse	GTAGATCTCAGCAAACTCGC	qRT-PCR
Actin-Forward	GTTGCACCACCTGAAAGGAAG	qRT-PCR internal control
Actin-Reverse	CAATGGGACTAAAACGCAAAA	qRT-PCR
GUS-Forward	ACGGCCTGTGGGCATTC	qRT-PCR
GUS-Reverse	GCGCTTTCCCACCAACG	qRT-PCR
PC-7-Forward	GTAGCGGTTGAGGTAGGA	generating constructs
PC-7-Reverse	GAGCACCTTGATAGATAAG	Generating constructs
PC29-Forward	TACCCGATCACCTTTCATG	generating constructs
PC29-Reverse	TGCACTCGCAGATGCCATC	generating constructs
34-Forward	CTGATTGGATTTGGATCACAACC	generating constructs
30-Reverse	TCAGCTTCGTTACAGGCGA	generating constructs

PC29-Forward	TACCCGATCACCTTTCATG	Resequence Ghd8
PC29-Reverse	TGCACTCGCAGATGCCATC	Resequence Ghd8
PC-3-Forward	AGTGGCATTGATGCACTGC	Resequence Ghd8
PC-3-Reverse	CAGGCTTCTTATGTTTCAC	Resequence Ghd8
PC-7-Forward	GTAGCGGTTGAGGTAGGA	Resequence Ghd8
PC-7-Reverse	GAGCACCTTGATAGATAAG	Resequence Ghd8
PC34-Forward	CTGATTGGATTTGGATCACAACC	Resequence Ghd8
PC30-Reverse	TCAGCTTCGTTACAGGCGA	Resequence Ghd8
PC33-Forward	TCCGAATGTCTAGTTCCCCACCG	Resequence Ghd8
PC55CR-Reverse	CATCAGCTTCGTTACAGGCGAG	Resequence Ghd8
PGhd8F1	AAAGGATCCGTGAAGCTAACAACACTAGC	truncated promoter
PGhd8R1	AAAAAGCTTTGCACTCGCAGATGCCATC	truncated promoter
PGhd8R2	AAAAAGCTTTTTCAACCTTAAGCTTCTG	truncated promoter
PNGhd8R4	AAAAAGCTTCTGCCTTTTCCTTTTATGAAAATGCAC	truncated promoter
PZGhd8R3	AAAAAGCTTCTGCCTTTTCCTTTGATG	truncated promoter
